# The osteology, taxonomy, and phylogenetic affinities of the Early Jurassic plesiosaur *Lusonectes sauvagei*

**DOI:** 10.7717/peerj.20611

**Published:** 2026-02-02

**Authors:** Sven Sachs, Daniel Madzia

**Affiliations:** 1 Abteilung Geowissenschaften, Naturkunde-Museum Bielefeld, Bielefeld, Germany; 2 Department of Evolutionary Paleobiology, Institute of Paleobiology, Polish Academy of Sciences, Warsaw, Poland

**Keywords:** Plesiosauria, Toarcian, Jurassic, Osteology, Phylogeny reconstruction, Portugal

## Abstract

The transition from the Early to the Middle Jurassic was marked by significant restructuring of plesiosaur communities. While knowledge of the earliest Middle Jurassic plesiosaurs is generally limited, Toarcian plesiosaur occurrences are abundant, though the vast majority of specimens have been unearthed in the United Kingdom and Germany. Here, we reassess *Lusonectes sauvagei*, an early-diverging plesiosaur from the lower to middle upper Toarcian of the São Gião Formation in Portugal. Originally described as *Plesiosaurus* sp., it was later established as a distinct taxon closely related to taxa currently encompassed within Microcleididae. Our firsthand examination of the holotype of *L. sauvagei* resulted in differing interpretations of certain aspects of its morphology, prompting a detailed osteological, taxonomic, and phylogenetic reevaluation. We provide a redescription of *L. sauvagei*, propose a new diagnosis, and investigate its phylogenetic affinities. Although the specimen is fragmentary and poorly preserved, our study suggests that, contrary to the original interpretation, *L. sauvagei* is not affiliated with *Microcleidus* spp. The taxon remains problematic and may represent either an early-diverging pliosaurid or a plesiosauroid. *Lusonectes* is one of the few diagnosable plesiosaurs from the upper Lower Jurassic found outside the classic British and German localities and thus offers insights into the diversity of plesiosaurs just prior to a major event in the evolutionary history of the clade.

## Introduction

During the transition from the Early to the Middle Jurassic (Toarcian–Bajocian), plesiosaur communities experienced notable restructuring of their composition (*e.g.*, Fischer, Weis & Thuy, 2021). This period saw the emergence and diversification of new clades, including cryptoclidian plesiosauroids and thalassophonean pliosaurids (Sachs, Abel & Madzia, 2023; Sachs, Eggmaier & Madzia, 2024), which began to dominate marine ecosystems. In turn, some older lineages, such as microcleidids, disappeared entirely, while others, like rhomaleosaurids, began to decline and gradually vanish from the fossil record, ultimately going extinct in the Callovian (late Middle Jurassic) (*e.g.*, Gasparini, 1997; Sato & Wu, 2008; Benson, Zverkov & Arkhangelsky, 2015a; Sachs, Abel & Madzia, 2022).

While knowledge of earliest Middle Jurassic plesiosaurs is generally limited, with only a handful of specimens on record (*e.g.*, Vincent, Bardet & Morel, 2007; Vincent et al., 2013; Rogov et al., 2019), Toarcian plesiosaur occurrences are abundant, particularly from European deposits. A significant number of specimens have been reported especially from the United Kingdom (see, *e.g.*, Benson et al., 2011a; Benson, Evans & Druckenmiller, 2012; Benton & Taylor, 1984; Brown, Vincent & Bardet, 2013; Forrest, 2000; Seeley, 1865; Smith & Benson, 2014; Smith & Lomax, 2019; Taylor, 1992; Owen, 1865; Watson, 1909; Watson, 1910; Watson, 1911) and Germany (*e.g.*, Dames, 1895; Fraas, 1910; Großmann, 2007; von Huene, 1923; Maisch & Rücklin, 2000; Marx et al., 2025; O’Keefe, 2001; O’Keefe, 2004; Sachs, Hornung & Kear, 2016a; Sachs, Abel & Madzia, 2023; Sachs, Eggmaier & Madzia, 2024; Sachs et al., 2025; Sachs & Madzia, 2025; Smith & Vincent, 2010; Stumpf, 2016; Vincent, 2010; Vincent, 2011; Vincent et al., 2017). Elsewhere, only relatively few specimens have been described, including material from France (Bardet, Godefroit & Sciau, 1999; Brignon, 2025; Sciau, Crochet & Mattei, 1990), Luxembourg (Vincent et al., 2019), Switzerland (Wild, 1968), or Russia (Rogov et al., 2019; Zverkov, Grigoriev & Danilov, 2021) and Australia (Thulborn & Warren, 1980; Kear, 2012). Many of these occurrences, however, are in need of thorough restudies. Others, in turn, represent important yet problematic specimens known from highly incomplete or poorly preserved material that may be prone to differing interpretations.

Here, we provide a reassessment of one such specimen, MG 33, the holotype of *Lusonectes sauvagei* Smith, Araújo & Mateus, 2012. *Lusonectes* is an early-diverging plesiosaur from the lower to middle upper Toarcian beds of the São Gião Formation near Alhadas, District of Coimbra, Portugal. The taxon was established based on a partial, poorly preserved skull, which includes an incomplete mandible. The specimen was initially described by French paleontologist Henri Émile Sauvage, who referred to it as *Plesiosaurus* sp. (Sauvage, 1897–1898). This classification was later followed by Bardet et al. (2008) and Ruiz-Omeñaca et al. (2009). However, Smith, Araújo & Mateus (2012), upon closer examination, recognized its uniqueness and named it *Lusonectes sauvagei*, placing it in Plesiosauridae.

During personal examination of the specimen we made several observations that differed from those of Smith, Araújo & Mateus (2012), warranting publication of the osteological, taxonomic, and phylogenetic reassessment of the taxon.

We provide a redescription of MG 33, illustrate it, evaluate its taxonomic significance, and explore its phylogenetic affinities, taking into account clearly observable features as well as those that are likely present but cannot be confirmed with certainty.

## Methods

### Phylogenetic analyses

**Data sampling.** We assessed the phylogenetic affinities of *Lusonectes sauvagei* using the matrix from Sachs et al. (2025), which represents a significantly revised version of the dataset originally compiled by Benson & Druckenmiller (2014). Apart from the inclusion of scores obtained from MG 33, the type specimen of *L. sauvagei*, we have also modified the scores of the holotype (SMNS 16812), and added the referred specimen (MH 7), of *Plesiopterys wildi*. No further modifications were made. The final matrix consisted of 133 operational taxonomic units (OTUs) scored for 270 characters. Of these, 67 were set as ‘additive’ (or ‘ordered’), following Madzia, Sachs & Lindgren (2019).

To provide a thorough evaluation of the potential phylogenetic affinities of *Lusonectes sauvagei*, we tested its placement using two separate OTUs. The first one, dubbed herein as ‘conservative’, comprises the ‘basic’ set of features observable in the type specimen. The second one, dubbed ‘experimental’ includes additional scores based on possible interpretations of characters related to the anterior interpterygoid vacuity (characters 96, 97, 106, 107, and 108 that have not been included in the ‘conservative’ OTU).

**Protocol.** Our analyses were conducted in TNT 1.6 (Goloboff & Morales, 2023), using maximum parsimony as the optimality criterion. We performed two sets of analyses (employing ‘basic’ and additional scores for *L. sauvagei*; see above), each comprising three runs: the first run was based on equal weights (EW), and the remaining two used the implied weighting function (IW) with concavity parameters (*K*) set at 9 and 15. The early sauropterygian *Neusticosaurus pusillus* was designated as the outgroup in all analyses.

TNT settings have been the same for all analyses: we restricted the maximum number of most parsimonious trees to 200,000 using the command “hold 200000”, which we included directly to the TNT file. A ‘New Technology’ search was conducted incorporating 500 addition sequences and default settings for sectorial searches, ratchet, drift, and tree fusing. After obtaining results from these searches, we ran an additional ‘Traditional’ search with tree bisection-reconnection (TBR) branch-swapping, using trees stored in RAM.

For the EW analysis, we assessed Bremer support by employing TBR while retaining suboptimal trees with up to three additional steps. Nodal support for the implied-weighting analyses was evaluated using Symmetric Resampling, with a ‘Traditional’ search conducted over 1,000 replicates, a default change probability of 33, and results expressed as frequency differences (GC).

See Supplementary Information I for the full character list and Supplementary Information II and III for the TNT-executable files.

## Results

### Description and comparisons

**Figure 1:**
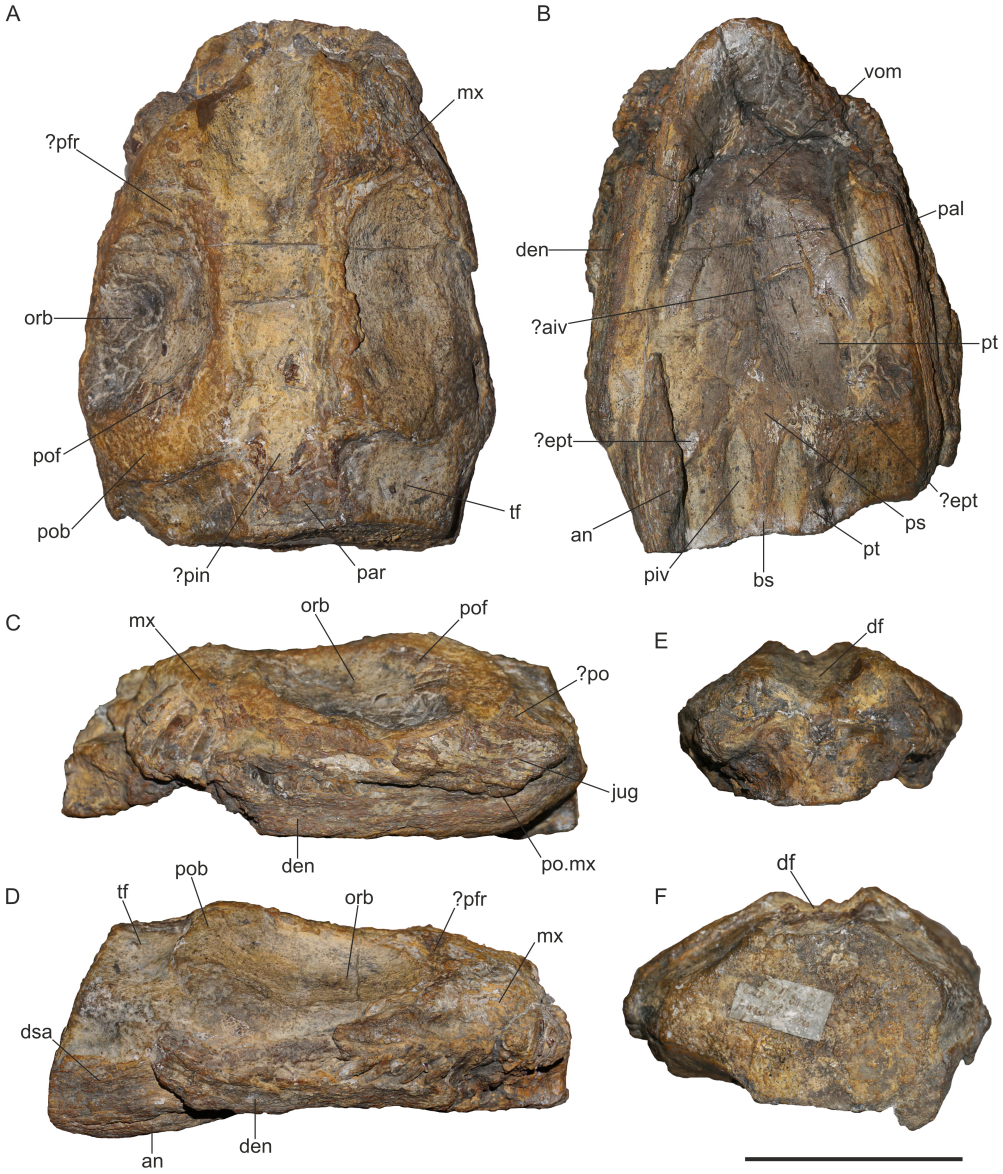
Holotype specimen of *Lusonectes sauvagei* (MG 33) in (A) dorsal, (B) central, (C) left lateral, (D) right lateral, (E) anterior, and (F) posterior view. Abbreviations: ?aiv, possible anterior interpterygoid vacuity; an, angular; bs, basisphenoid; den, dentary; df, dorsal furrow; dsa, dentary or surangular; ?ept, possibly ectopterygoid; jug, jugal; mx, maxilla; orb, orbit; pal, palatine; par, parietal; ?pin, possibly pineal foramen; piv, posterior interpterygoid vacuity; ?po, probably postorbital; pob, postorbital bar; po.mx, posterior end of maxilla; pof, postfrontal; ?prf, probably prefrontal; ps, parasphenoid; pt, pterygoid; tf, temporal fenestra; vom, vomer.

**Figure 2:**
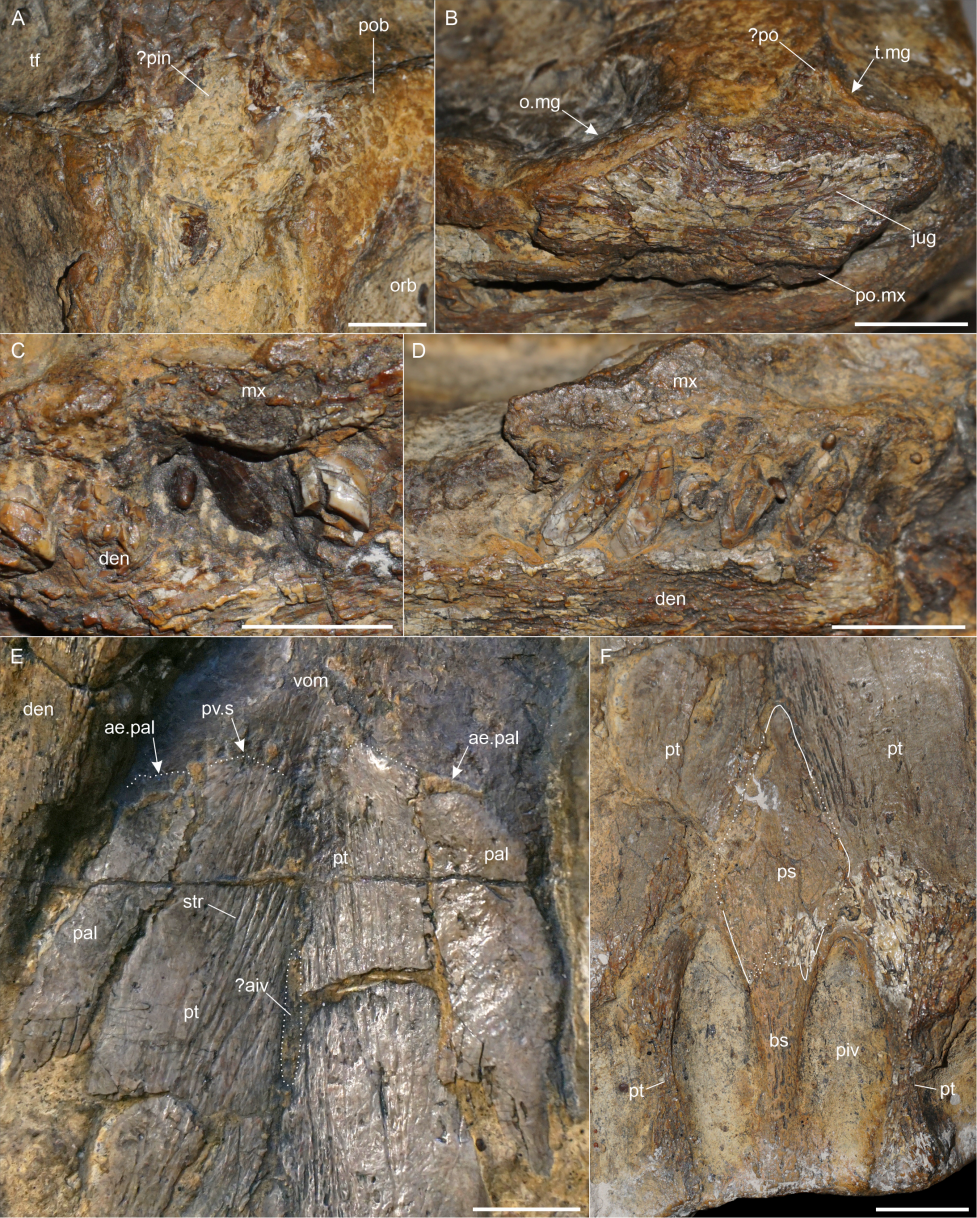
Anatomical details of *Lusonectes sauvagei* (MG 33). (A) posterior interorbital section showing the possible pineal foramen, (B) posterior maxilla, jugal and ?postorbital in lateral view, (C) tooth crowns showing the enamel, (D) damaged right maxillary and dentary teeth, (E) anterior pterygoids, showing the palatine and supposed vomer sutures, (F) posterior palatal section of the skull. Scale bars equal one cm. Abbreviations: ae.pal, anterior end of palatine; ?aiv, possible anterior interpterygoid vacuity; bs, basisphenoid; den, dentary; mx, maxilla; o.mg, margin of orbit; orb, orbit; pal, palatine; ?pin, possibly pineal foramen; piv, posterior interpterygoid vacuity; ?po, probably postorbital; po.mx, posterior end of maxilla; pob, postorbital bar; ps, parasphenoid; pt, pterygoid; pv.s, potential pterygoid-vomer suture; str, striations; t.mg, margin of temporal fenestra; tf, temporal fenestra; vom, vomers.

**General remarks.** MG 33 comprises the midsection of the skull and mandible (Fig. 1). Most of the preorbital portion and the posteriormost part are missing. The preservation of the specimen, in particular that of the dorsal side of the skull, is very poor and no sutures are traceable. The left orbit is nearly complete. It has an oval shape with the long axis in an anteroposterior direction (Fig. 2F). The right orbit is incomplete, missing the posterior and lateroventral margins (Fig. 1A). The postorbital bars are present on both sides but only the anterior margins of the temporal fenestrae are preserved (Figs. 1C, 1D). Smith, Araújo & Mateus (2012) mentioned that there is no pineal foramen. However, we think that the pineal foramen might have been present at the level of the temporal bar. A centrally placed depression is present at that region, which is filled with matrix and damaged, missing the anterior margin (Figs. 1A, 2A).

The preservation is much better from the ventral view which shows the morphology of the palate, with some traceable sutures. Smith, Araújo & Mateus (2012) noted that the medial margin of the left internal naris is visible as a ridge formed by the vomer. However, this part of the skull is damaged and we were unable to trace the structure with certainty.

Selected measurements of MG 33 are provided in Table 1. For original interpretations of elements and sutures, we refer to Smith, Araújo & Mateus (2012, fig. 1). The sutures that we interpret differently (or suggest a possible alternative interpretation for) are illustrated in Fig. 2.

**Table 1:** Selected measurements of the *Lusonectes sauvagei* holotype (MG 33).

Skull, maximum preserved length: 126 mm
Skull, maximum preorbital width: 81 mm
Skull, maximum postorbital width: 96 mm
Orbit (left), maximum length: 45 mm
Orbit (left), maximum width: 31 mm
Pterygoid (left), length from posterior end to supposed vomer contact: 88 mm
Pterygoid (left), width at supposed vomer contact: 10 mm
Pterygoid (right), length from posterior end to supposed vomer contact: 92 mm
Pterygoid (right), width at supposed vomer contact: 11 mm
Pterygoid (left), maximum width lateral to posterior interpterygoid vacuity: 8 mm
Pterygoid (right), maximum width lateral to posterior interpterygoid vacuity: 8 mm
?Anterior interpterygoid vacuity, length: 15 mm
?Anterior interpterygoid vacuity, maximum width: 3 mm
Posterior interpterygoid vacuity (left), length: 21 mm
Posterior interpterygoid vacuity (left), maximum width: 8 mm
Posterior interpterygoid vacuity (right), length: 24 mm
Posterior interpterygoid vacuity (right), maximum width: 7 mm
Parasphenoid, length of cultriform process: 25 mm
Basisphenoid, length as preserved: 20 mm
Basisphenoid, maximum width: 6 mm

**Maxilla.** The maxillae are badly damaged and partly exposed in dorsal and lateral views (Figs. 1A, 1C, 1D). The more complete left maxilla extends ventral to the jugal. The posterior end of the right maxilla is missing. Anterior to the orbit, the right maxilla is dorsoventrally high and would have contacted either the prefrontal or the frontal.

**Prefrontal and frontal.** On the left side of the skull, the curved anterodorsal edge of the orbit is well preserved. In other Early Jurassic plesiosaurs, this part of the orbit is usually formed by the prefrontal (see *e.g.*, Bardet, Godefroit & Sciau, 1999, fig. 3; Benson, Evans & Druckenmiller, 2012, fig. 4; Benson, Evans & Taylor, 2015b, fig. 1). The dorsal interorbital section is composed by either the prefrontals and frontals (as in *Microcleidus brachypterygius*, see Maisch & Rücklin, 2000, fig. 3) or by the frontals and the premaxillae that extend far posteriorly to contact the parietals (as in *Rhomaleosaurus cramptoni*, see Smith & Dyke, 2008, fig. 1). In MG 33, the dorsal interorbital section is indented (Fig. 1A). Even though the indention appears to be an artefact of the preservation, dorsally concave frontals have been reported for the Cretaceous plesiosauroid *Brancasaurus brancai* (Sachs, Hornung & Kear, 2016b).

**Postfrontal.** A small curved fragment, preserved at the posterodorsal edge of the left orbit (Figs. 1A, 1C), was identified as postfrontal by Smith, Araújo & Mateus (2012, fig. 1). The postfrontal is found at the same position in other Early Jurassic plesiosaurs, such as *Macroplata tenuiceps* (Ketchum & Smith, 2010, fig. 1) or *Stratesaurus taylori* (Benson, Evans & Taylor, 2015b, fig. 1). It participates in the orbital margin as in most plesiosaurs (see Benson & Druckenmiller, 2014, appendix 2, character 36).

**Postorbital.** Part of the left postorbital appears to be present dorsal to the jugal, as likewise identified by Smith, Araújo & Mateus (2012). The fragment includes a section of the original anterolateral margin of the left temporal fenestra (Figs. 1C, 2B). In other Early Jurassic plesiosaurs, such as *Seeleyosaurus guilelmiimperatoris* (Sachs et al., 2025), *Plesiosaurus dolichodeirus* (Storrs, 1997), or *Atychodracon megacephalus* (Cruickshank, 1994), this margin is formed by the postorbital. Smith, Araújo & Mateus (2012, fig. 1B) noted that part of the postorbital is also attached to the fragmentary postfrontal but there is no visible suture so we cannot confirm this observation. A small patch on the right side of the skull was likewise identified as possibly representing part of the postorbital by Smith, Araújo & Mateus (2012, fig. 1A). We think that it might be part of either the postorbital or the jugal.

**Jugal.** The left jugal is preserved as an anteroposteriorly elongate element that is placed posterolateral to the left orbit (Figs. 1C, 2B). Smith, Araújo & Mateus (2012) mentioned that the jugal formed part of the orbital margin, a condition found in most rhomaleosaurids (see Benson & Druckenmiller, Appendix 2, character 37), the early-diverging pliosaurids *Thalassiodracon hawkinsii* (Benson et al., 2011a) and *Attenborosaurus conybeari* (Benson & Druckenmiller, Appendix 2, character 37) and the plesiosauroid *Plesiosaurus dolichodeirus* (Storrs, 1997), among Early Jurassic taxa. However, there is a distance of four mm between the anteroventrally-inclined anterior edge of the jugal and the margin of the orbit. Furthermore, the anterior portion of the jugal bears a distinct zig-zag-shaped pattern which could be part of a jugal-maxillary suture. If so, the jugal would have been excluded from the orbit, as in *Microcleidus homalospondylus* (Brown, Vincent & Bardet, 2013).

**Parietals.** Only parts of the parietals are present (Fig. 1A). They formed the separation of the temporal fenestrae, as shown by Smith, Araújo & Mateus (2012, fig. 1C).

**Vomers.** Only the posterior sections of the vomers are exposed (Figs. 1A, 2E), but their sutural contact with the pterygoids cannot be traced with certainty. A posterolateral contact with the palatines was illustrated by Smith, Araújo & Mateus (2012, fig. 1D) but we were unable to confirm such a condition.

**Palatines.** The palatines are partly exposed in ventral view (Figs. 1B, 2E). They are separated from one another by the pterygoids. A regular medial suture to the latter is visible over the entire preserved length of the palatines. Their lateral contacts with the maxillae are obscured by matrix and other elements. The palatines have a slightly convex ventral surface. Smith, Araújo & Mateus (2012, fig. 1D) reconstructed the palatines to extend far anteriorly and having a long suture with the vomers, but we were able to follow the palatines only to the level of the supposed pterygoid-vomer contact (Fig. 2E).

**Pterygoids.** Both pterygoids are nearly complete (Fig. 1B). The anterior sutures cannot be traced with certainty, but a structure resembling an irregular and transverse broad pterygoid–vomer suture is present on both sides (Fig. 2E). Smith, Araújo & Mateus (2012, fig. 1D) illustrated the sutures approximately at the same position but indicated the conjoined anterior pterygoids to be pointed. A broad pterygoid-vomer contact is found in some Early Jurassic plesiosaurs, such as the rhomaleosaurids *Meyerasaurus victor* (Smith & Vincent, 2010, fig. 2), *Atychodracon megacephalus* (Cruickshank, 1994, fig. 4), or *Rhomaleosaurus cramptoni* (Smith & Dyke, 2008, fig. 1), the pliosaurids *Thalassiodracon hawkinsii* (Benson et al., 2011a), *Hauffiosaurus tomistomimus* (Benson et al., 2011b), and in the early-diverging plesiosauroid *Plesiosaurus dolichodeirus* (Storrs, 1997). Anterolaterally, the pterygoids contact the palatines with a regular slightly anteromedially inclined suture. The anterior pterygoid portion is depressed at the midline. It is uncertain whether this is a genuine condition or taphonomic. A straight midline suture connects the pterygoids anteriorly. The suture is interrupted approximately in its mid-section by an elongate and transversely narrow depression or opening that is partly broken and largely covered by matrix. Even though the preservation does not allow the structure to be assessed without remaining doubts, it may potentially represent the anterior interpterygoid vacuity (Figs. 1B, 2E). A similar, slit-shaped, anterior interpterygoid vacuity that is bound by pterygoids only is found in the pliosaurids *Cryonectes neustriacus* (Vincent et al., 2013, fig. 4) and *Hauffiosaurus tomistomimus* (Benson et al., 2011b, fig. 5). Smith, Araújo & Mateus (2012) mentioned that *Lusonectes sauvagei* lacks an anterior interpterygoid vacuity. Posterior to the potential anterior interpterygoid vacuity, the pterygoids are connected to the point where the cultriform process of the parasphenoid separates them. A remnant of the supposed left ectopterygoid (as interpreted by Smith, Araújo & Mateus, 2012) contacts the pterygoid approximately at the level of the anterior margins of the posterior interpterygoid vacuities (Fig. 1B). It remains, however, unclear if this element is the ectopterygoid or part of the pterygoid (see discussion below). Posteriorly, the pterygoids extend adjacent to the posterior interpterygoid vacuities of which they form the anterior and lateral margins (Fig. 2F).

**?Ectopterygoid.** A small, poorly preserved element, placed lateral to the left pterygoid at the level of the anterior edges of the posterior interpterygoid vacuities, was interpreted as the ectopterygoid by Smith, Araújo & Mateus (2012, fig. 1D) (Fig. 1B). However, the condition of this element does not permit any meaningful description, and the suture to the pterygoid is not visible. At the same position, a fragment is also present on the right side of the skull (Fig. 1B). The placement is similar to where the ectopterygoid is found, for example, in *Microcleidus brachypterygius* (Maisch & Rücklin, 2000, fig. 4) or *Atychodracon megacephalus* (Cruickshank, 1994, fig. 4). However, some taxa, such as *Stratesaurus taylori*, have lateral pterygoid wings that are likewise found at a similar position (see Benson, Evans & Taylor, 2015b, fig. 23). As such, it remains unclear whether these elements represent the ectopterygoids or parts of the pterygoids.

**Parasphenoid.** The parasphenoid is complete and well preserved, only the lateral sutural contacts to the pterygoids are partly damaged. A prominent cultriform process is formed. It is flat and arrowhead-shaped (Figs. 1B, 2F). The cultriform process separates the pterygoids, and extends further anteriorly than in most Early Jurassic plesiosaurs, such as the microcleidid *Microcleidus homalospondylus* (Brown, Vincent & Bardet, 2013, fig. 1), the pliosaurid *Cryonectes neustriacus* (Vincent et al., 2013, fig. 4) or the rhomaleosaurid *Atychodracon megacephalus* (Cruickshank, 1994, fig. 4) where a considerably shorter cultriform process is present. An equally long cultriform process has been described for *Plesiosaurus dolichodeirus* (Storrs, 1997). Around the anterior one-fourth of the posterior interpterygoid vacuities, an irregular parasphenoid-basisphenoid suture is formed. The suture corresponds largely to the condition illustrated by Smith, Araújo & Mateus (2012, fig. 1D). However, it is not as distinct as other sutures observable on the ventral aspect of the skull. A similar, short parasphenoid that extends only to the anterior section of the posterior interpterygoid vacuities is found in *Thalassiodracon hawkinsii* (Benson et al., 2011a), *Cryonectes neustriacus* (Vincent et al., 2013), *Hauffiosaurus* spp. (Benson et al., 2011b), and *Plesiosaurus dolichodeirus* (Storrs, 1997).

**Basisphenoid.** The basisphenoid meets the parasphenoid *via* an irregular suture (Figs. 1B, 2F). It forms most of the medial and posterior borders of the posterior interpterygoid vacuities. The surface of the basisphenoid is flat and only slightly dorsoventrally thickened anteriorly. There is no distinct keel, which resembles the condition in the pliosaurid *Thalassiodracon hawkinsii* (Benson et al., 2011a) and in the plesiosauroids *Microcleidus brachypterygius* (Maisch & Rücklin, 2000) and *Plesiopterys wildi* (O’Keefe, 2004). Posteriorly, the basisphenoid widens transversely, becoming flatter where the basisphenoid forms the posterior margin of the posterior interpterygoid vacuities (Fig. 2F).

**Mandible.** The mandible is only partly preserved and severely broken (Figs. 1B–1D). The dentary is visible in lateral view on both sides. However, it lacks the anterior portion including the symphysis. On the right side, there is a posterodorsal fragment that has been identified as the surangular by Smith, Araújo & Mateus (2012). The mandible is damaged at this section and the fragment has been shifted anteromedially. Additionally, it is partly obscured by the dentary and matrix (Fig. 2D). For that reason, it remains unclear whether this fragment represents the anterior surangular portion or the posterior dentary. In contrast, we confirm the presence of the distinct, lateroventrally-positioned angular suture observed by Smith, Araújo & Mateus (2012, fig. 1). The angular is visible in both mandibular rami and the suture allows designating the separation between the angular and the dentary. The anterior extends of the angulars cannot be assessed because they are damaged on both sides. For the same reason it cannot be judged whether any of the preserved mandibular elements is the splenial, as depicted by Smith, Araújo & Mateus (2012, fig. 1). The anteriormost preserved part of the mandible is transversely curved which, however, may be taphonomic.

**Dentition.** The teeth are slender, pointed, and have a circular cross-section (Figs. 2C, 2D). There are no apparent signs of anisodonty or heterodonty. Most teeth are damaged and the crowns are often vertically sliced through so that the pulp cavity is exposed (Fig. 2D). A few crowns preserve the enamel, which lacks any ornamentations (Fig. 2C) Smith, Araújo & Mateus (2012: p. 261) noted that the enamel surface appears to be entirely smooth and unornamented, recognizing that it may be a preservational artefact though they did not rule out the possibility that the condition is, in fact, genuine, which could, according to the authors, represent a diagnostic character. We consider this option highly unlikely and treat the lack of ornamentation as being due to preservation.

### Results of phylogenetic analyses

**Figure 3:**
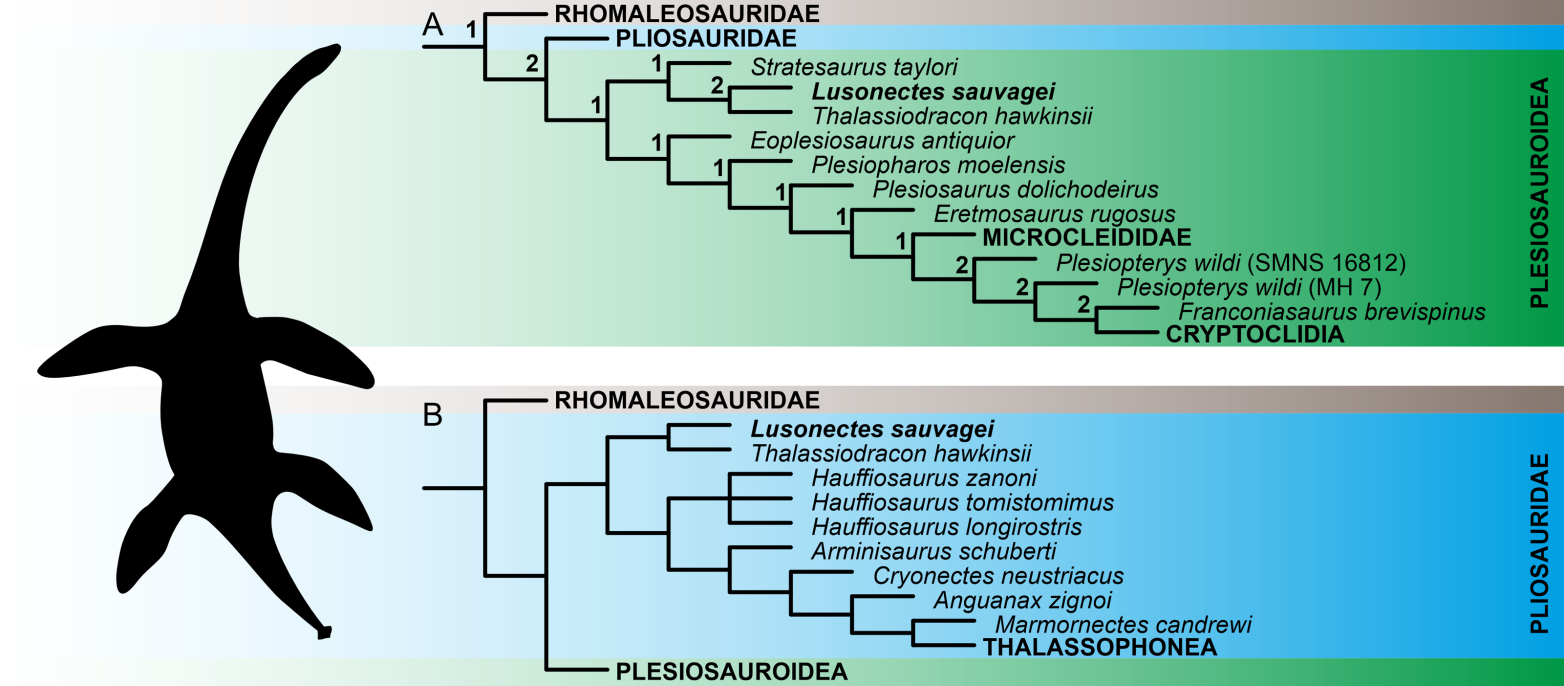
Reconstruction of the phylogenetic placement of *Lusonectes sauvagei* (MG 33) among early-diverging plesiosaurs. (A) Reduced strict consensus tree inferred through parsimony analyses using equal weighting (numbers at nodes indicate the Bremer support values) and (B) reduced strict consensus tree inferred through weighted parsimony analyses with *K* set to 9 and 15. Both parsimony analyses using equal weighting and all four analyses using implied weighting (regardless of whether they utilized the ‘conservative’ or ‘experimental’ operational taxonomic units; see main text for details) inferred the same general tree topologies.

The numerical results of our phylogenetic analyses (the numbers of most parsimonious trees (MPTs) and their ‘best scores’, and Consistency and Retention indices) are provided in Table 2. Reduced tree topologies, focusing on the inferred placement of *Lusonectes sauvagei*, are visualized on Fig. 3. For full tree topologies, with the nodal support values, see Supplementary Information IV.

The analyses of both ‘conservative’ and ‘experimental’ OTUs (see Methods for details) resulted in broadly similar tree topologies, indicating that the inclusion of cautiously interpreted character states related to the anterior interpterygoid vacuity did not significantly impact the inferred tree topologies. All weighted parsimony analyses, regardless of whether we used the ‘conservative’ or ‘experimental’ OTU, and irrespective of the selected *K*-value, reconstructed *L. sauvagei* as an early-diverging pliosaurid closely related to *Thalassiodracon hawkinsii*. The analyses based on equal weights reconstructed the clade formed by the two species as well but preferred assignment among early plesiosauroids.

### Systematic paleontology

**Table:** 

Plesiosauria De Blainville, 1835
*Lusonectes* Smith, Araújo & Mateus, 2012
*Lusonectes sauvagei* Smith, Araújo & Mateus, 2012

**Type specimen.** MG 33, a partial skull, including incomplete mandible.

**Type locality and horizon.** Near Alhadas, District of Coimbra, Portugal; São Gião Formation, lower to middle upper Toarcian, Lower Jurassic.

**Table 2:** Numerical results of the phylogenetic analyses.

**Run**	**MPT (NT)**	**BS**	**MPT (TS)**	**CI**	**RI**
**EW** ***c***	30	2,103	200,000	0.190	0.687
**IW** ***c*** **(** ***K*** **= 9)**	15	109.91044	96,957	0.188	0.684
**IW** ***c*** ** (** ***K*** **= 15)**	30	78.56899	23,085	0.189	0.685
**EW** ***e***	28	2,103	200,000	0.190	0.687
**IW** ***e*** ** (** ***K*** **= 9)**	11	109.91044	96,957	0.188	0.684
**IW** ***e*** ** (** ***K*** **= 15)**	27	78.56899	23,085	0.189	0.686

**Notes.**

BS best score (tree length) CI Consistency Index EW parsimony analysis using equal weighting IW parsimony analysis using implied weighting MPT number of most parsimonious trees NT ‘New Technology’ search RI Retention Index TS ‘Traditional’ search

Lowercase ‘*c*’ and ‘*e*’ indicate analyses in which we used ‘conservative’ and ‘experimental’ OTUs of *Lusonectes sauvagei*, respectively.

**Revised diagnosis** (autapomorphy marked with *). Parasphenoid short, extending to the anterior fourth of the posterior interpterygoid vacuities; cultriform process flat and equal in length with the posterior interpterygoid vacuities, incising the pterygoids*; basisphenoid lacks a distinct midline ridge, lowers and at the same time widens posteriorly; basisphenoid forms most of the medial margin of the posterior interpterygoid vacuities.

## Discussion

Smith, Araújo & Mateus (2012) provided a detailed diagnosis for *Lusonectes sauvagei*, and identified one autapomorphy and a unique combination of character states. Below, we reevaluate each of the character states (see also Table 3).

**Table 3:** Differences in the interpretation of the anatomy of *Lusonectes sauvagei* between Smith, Araújo & Mateus (2012) and the present study.

**Original interpretations (Smith, Araújo & Mateus, 2012)**	**Present study**
No indication of a pineal foramen	Part of the pineal foramen might be present at the level of the temporal bar
Medial margin of the left internal naris present as a ridge formed by the vomer	This structure could not be traced with certainty because of poor preservation
Prefrontal not identified	Prefrontal potentially present at the anterodorsal edge of the left orbit
Small patch on the right side of the skull possibly represents a part of the postorbital	This might be a part of either the postorbital or the jugal
Jugal participates in framework of orbit	Unclear whether jugal participates or whether the maxilla precluded the jugal from orbit
Posterolateral vomer-palatine contact present	Cannot be confirmed
Palatines extend far anteriorly and contact the vomers	Palatines likely extend less far anteriorly
Conjoined anterior pterygoids pointed	Anterior end of each pterygoid appear more transversely rounded
Anterior interpterygoid vacuity absent	Uncertain but anterior interpterygoid vacuity possibly present
Right surangular present	Unclear whether this portion is the anterior surangular or the posterior dentary
Right splenial present	Unclear because of poor preservation
Teeth have no ornamentation or striations, possibly be due to abrasion	Lack of ornamentation is highly unlikely; condition is preservational

*Broad triangular cultriform process of parasphenoid that is as long as the posterior interpterygoid vacuities*. Smith, Araújo & Mateus (2012) considered the shape and extent of the cultriform process as an autapomorphy of *Lusonectes sauvagei*. We confirm such an appearance of the cutriform process which is, indeed, flat, broad, and has a triangular shape. The more complete right posterior interpterygoid vacuity measures 24 mm in length, the cultriform process from the anteriormost corner to anterior edge of the left posterior interpterygoid vacuity margin measures 25 mm. We agree that this morphology is unusual and autapomorphic for *Lusonectes sauvagei*. As discussed above, a flat cultriform process is present in other plesiosaurs as well, but here they are not as long as in *Lusonectes*. Also, the posterior interpterygoid vacuities are usually longer than the cultriform process in other plesiosaurs (compare, *e.g.*, Cruickshank, 1994, fig. 4, Smith & Vincent, 2010, fig. 2, Brown, Vincent & Bardet, 2013, fig. 1, Vincent et al., 2013, fig. 4).

*Jugal contacts the orbital margin.* We were unable to confirm this condition. As outlined in the description, it is not clear if the anterior edge of the jugal is genuine or damaged, and whether a jugal-maxillary suture extended alongside the preserved anterior jugal margin.

*A distinct parasphenoid–basisphenoid suture exposed between the posterior interpterygoid vacuities*. We agree and consider this condition likewise to be diagnostic. Compared with the other sutures visible on the ventral side of the skull, the parasphenoid–basisphenoid suture is not as distinctive.

*Unkeeled ventral parabasisphenoid with a flat anterior and gently convex posterior region of the ventral surface.* A flat surface within the posterior interpterygoid vacuity is widespread and present in various plesiosaurs (see the distribution of character state 83.1 in Benson & Druckenmiller, 2014). Among Early Jurassic taxa, a similar flat surface was described for the pliosaurid *Thalassiodracon hawkinsii* (Benson et al., 2011b), and for the plesiosauroids *Microcleidus brachypterygius* (Maisch & Rücklin, 2000) and *Plesiopterys wildi* (O’Keefe, 2004). Nevertheless, the ventral side of the basisphenoid, which forms the posterior section of the parabasisphenoid, is actually convex anteriorly and flattened posteriorly.

*Lack of an anterior interpterygoid vacuity*. We observed a slit-like structure that may potentially represent the anterior interpterygoid vacuity.

*Palatal striations on the ventral surface of the pterygoids*. We confirm the presence of distinctive palatal striations. However, these structures are present in some plesiosaur specimens (see, *e.g.*, Sachs, Hornung & Kear, 2017, fig. 3; Sachs & Kear, 2017, fig. S3). In many other taxa the presence of such striations cannot be confirmed due to the preservation. For that reason, it is difficult to say how widespread these structures are and whether, or to what degree, they are taxonomy-informative.

*The teeth have no ornamentation or striations, but this may be due to abrasion*. The teeth are poorly preserved. The enamel is visible only in a few teeth, and, indeed, these do not show any ornamentation. However, as noted above, we are convinced this condition does not reflect the genuine appearance of the tooth crowns.

### Is *Lusonectes sauvagei* a pliosaurid, a plesiosauroid, or a rhomaleosaurid?

The early evolution of plesiosaurs is contentious, with rootward branching being highly susceptible to taxon sampling and the selection of tree search strategies. This instability has led to alternative hypotheses regarding the interrelationships of the three major plesiosaur clades (pliosaurids, plesiosauroids, and rhomaleosaurids) as well as the placement of numerous early-diverging plesiosaur taxa, including *Anningasaura lymense*, *Stratesaurus taylori*, and *Thalassiodracon hawkinsii* (*e.g.*, Fischer et al., 2018; Madzia & Cau, 2020; Puértolas-Pascual et al., 2021; Sachs, Eggmaier & Madzia, 2024; Sachs et al., 2025; see also Supplementary Information IV of the present study for alternative placements of some early-diverging taxa resulting from parsimony analyses using equal and implied weighting). Some of these taxa (notably *Thalassiodracon hawkinsii*) have been inferred here as potential close relatives of *Lusonectes sauvagei*.

In the original description, Smith, Araújo & Mateus (2012) reconstructed *Lusonectes sauvagei* among plesiosauroids, as part of a branch comprising *Occitanosaurus*, *Hydrorion*, and *Microcleidus*. The type species of *Occitanosaurus* (*O. tournemirensis*) and *Hydrorion* (*H. brachypterygius*) are currently commonly regarded as referable to *Microcleidus* (Benson, Evans & Druckenmiller, 2012), and the clade itself would approximate Microcleididae *sensu* Benson, Evans & Druckenmiller (2012). Our analyses based on equal weights reconstructed *Lusonectes* as a plesiosauroid as well. However, they placed it within an earliest-diverging clade additionally comprising *Thalassiodracon* and *Stratesaurus*, two taxa with partly unstable phylogenetic ties. The weighted parsimony analyses, in turn, placed *Lusonectes* and *Thalassiodracon* among pliosaurids, while reconstructing *Stratesaurus* as the earliest-diverging rhomaleosaurid.

Simulation-based studies suggest that weighted parsimony analyses outperform analyses based equal weighting (Goloboff, Torres & Arias, 2018), although this might not work universally, especially when dealing with smaller character matrices involving <50 terminals (Ezcurra, 2024). While we are inclined to treat *Lusonectes* to be possibly affiliated with *Thalassiodracon* the two alternative placements of *L. sauvagei* are likely equally plausible at the moment.

### Concluding remarks

Although *L. sauvagei* is based on highly fragmentary and poorly preserved specimen (MG 33), it represents a diagnosable plesiosaur taxon that can be distinguished from other early-diverging plesiosaurs by one autapomorphy and a unique combination of character states, especially those observed on the ventral aspect of the skull.

Our comparisons, supplemented with two sets of phylogenetic analyses, enabled us to identify numerous similarities especially with early-diverging pliosaurids. Owing to the fragmentary nature of MG 33 and its poor preservation, we emphasize that the results of our phylogenetic analyses should be treated with considerable caution and viewed as merely supplementary to our comparisons, providing additional insight into the phylogenetic placement of the taxon. Still, the originally-inferred association with microcleidid plesiosauroids appears to be unlikely.

Additionally, our first-hand inspection of MG 33 and a point-by-point evaluation of the observations made by Smith, Araújo & Mateus (2012) resulted in a number of differing interpretations. More importantly, however, we were also able to confirm the presence of the autapomorphy identified by Smith, Araújo & Mateus (2012), thus supporting the distinctive nature of the taxon.

Although *Lusonectes sauvagei* is difficult to study and remains poorly understood, it is one of the few diagnosable plesiosaur taxa from the Toarcian, discovered outside the ‘classic’ localities in the United Kingdom and Germany. It represents an important taxon contributing to our understanding of the diversity and disparity of plesiosaurs shortly before a major restructuring of marine reptile faunas at the transition of the Early and Middle Jurassic.

##  Supplemental Information

10.7717/peerj.20611/supp-1 Supplemental Information 1 Character list for phylogenetic analyses of Plesiosauria

10.7717/peerj.20611/supp-2 Supplemental Information 2 Matrix 1 executable in TNT

10.7717/peerj.20611/supp-3 Supplemental Information 3 Matrix 2 executable in TNT

10.7717/peerj.20611/supp-4 Supplemental Information 4 Extended results of phylogenetic analyses

## Institutional abbreviations

MG: Museu Geológico, Lisbon Portugal
MH: Urwelt-Museum Hauff, Holzmaden, Germany

## Additional Information and Declarations

### Data Availability

The following information was supplied regarding data availability: The character matrices necessary to replicate the phylogenetic analyses are available in the Supplementary Files.
