# Supplementary material for: The osteology, taxonomy, and phylogenetic affinities of the Early Jurassic plesiosaur Lusonectes sauvagei
Source: PeerJ. 2026 Feb 2;14:e20611. doi: 10.7717/peerj.20611 (PMC12875220; doi:10.7717/peerj.20611)
Supplement: Supplemental Information 1 [file peerj-14-20611-s001.docx]

**Supplementary Information I**

**The osteology, taxonomy, and phylogenetic affinities of the Early Jurassic plesiosaur *Lusonectes sauvagei***

Sven Sachs and Daniel Madzia

**Character list for the phylogenetic analyses of Plesiosauria**

**1.** **Transverse constriction of the rostrum at the premaxilla-maxilla suture:** absent (0); present (1).

**2.** **Maxilla, lateral expansion of maxilla posterior to maxilla-premaxilla suture accommodates expanded caniniform bases [‘roots’]:** absent (0); present (1).

**3.** **Ratio of orbit length in dorsal view to temporal fenestra length:** 0.3–0.7 (0); >0.8 (1).

**4.** **Ratio of pre-orbital skull length to total skull length measured in dorsal view:** <0.44 (0); 0.45–0.55 (1); >0.56 (2).

**5.** **Orbit, ventral margin of outline in lateral view:** concave, resulting in a suboval orbital outline (0); convex, resulting in reniform orbital outline with prominent lobate anterior extension (1).

**6.** **Dorsal margin of orbit, outline in dorsolateral view:** concave, forming part of a suboval orbit (0); convex, skull roof projects into orbit (1).

**7.** **Relative skull length compared to length of dorsal series:** 0.20–0.30 (0); 0.31–0.39 (1); >0.40 (2).

**8.** **Inclination of the suspensorium:** sub-vertical or weakly inclined (~80–90º) (0); significantly inclined (<70º) (1).

**9.** **Relative positions of external and internal nares:** internal naris posterior to external naris (0); nares overlap (1); internal naris anterior to external naris (2).

**10.** **Position of the mandibular glenoid fossa:** coplanar with the occipital condyle (0); just posterior to the occipital condyle (1); far posterior to occipital condyle, distance at least equal to basicranial (basioccipital+basisphenoid) length (2).

**11.** **Fluted ornamentation of bone surface around the dorsal orbit margin (on prefrontal or postfrontal):** absent (0); striations oblique to orbit margin (1); striations perpendicular to orbit margin (2).

**12.** **Temporal bar, suborbital margin:** smoothly curved (0); squared-off posteroventral margin of suborbital skull forms abrupt edge (1).

**13.** **Alveolar margin of upper jaw in lateral view:** approximately straight or weakly convex (0); undulating, forming 'scalloped' margin (1).

**14.** **Premaxilla, external surfaces:** marked neurovascular foramina but otherwise smooth (0); numerous sharp rugose crests (1); consistently undulating with rounded ridges (2).

**15.** **Premaxilla contact along the dorsal midline:** contacts anterior extension of frontals only (0); partially overlaps the frontal along the midline (1); overlaps the entire length of the frontal along the dorsal midline and contacts the parietal (2).

**16.** **Premaxilla, posterior termination:** tapering and non-interdigitating or weakly interdigitating (0); broad, deeply interdigitating suture with the frontal or parietal (1).

**17.** **Premaxilla, dorsomedian ridge:** absent or indistinct (0); prominent, forming either a narrow crest, a broad bar-like ridge, or a mound-like eminence on the dorsomedian surface of the rostrum (1).

**18.** **Premaxilla, morphology of dorsomedian ridge:** narrow and crest-like (taller than wide) (0); broad, occupying most of the internarial width of the rostrum (1); posterior mound (2).

**19.** **Premaxilla, dorsomedian ridge location:** anterior (0); posterior (1); elongate, extends from interorbital region to rostral tip (2).

**20.** **Premaxilla dorsomedian foramen:** absent (0); present (1).

**21.** **Premaxilla, participation in medial rim of external naris:** participates broadly along anteroposterior length of external naris (0); does not participate (1); small contact at anterodorsal corner of external naris (2).

**22.** **Premaxilla, constriction of posteromedian process at level of external naris:** absent (0); present, and does not expand to original width posterior to naris (1); present, but premaxilla expands to original width posterior to naris (2).

**23.** **Premaxilla-maxilla sutures:** converging posteromedially gradually, for entire length (0); anterior portion extends dorsomedially then abruptly curves posteriorly, resulting in a parallel-sided appearance of the posterior process of the premaxilla (1).

**24.** **Premaxilla-maxilla sutures, morphology anteriorly:** curved, but only weakly interdigitating, sinuous, or straight (0); pronounced, anteroposteriorly interdigitating contact with ‘zig-zag’ appearance (1).

**25.** **Maxilla and dentary, posterior extent of maxillary tooth row:** around orbital midlength or more anteriorly (0); ventral to postorbital bar (1); ventral to temporal fenestra midlength (2).

**26.** **Maxilla-squamosal contact:** absent (0); present (1).

**27.** **Maxilla participation in internal naris:** participates (0); does not participate (1).

**28.** **Maxilla, trough or depressed region anterior to external naris:** absent (0); present, defined laterally by a longitudinal ridge but does not extend far anteriorly (1); prominent longitudinal trough extends most of the prenarial length of the maxilla (2).

**29.** **Posteromedial extension of the maxilla:** extends to anteromedial margin of the external naris (0); extends to midpoint of the medial margin of the external naris (1); extends posteromedial to the external naris (2).

**30.** **Maxilla, posteromedial (=posterodorsal) portion:** not subdivided, forms simple sheet of bone (0); subdivided by anteroposteriorly oriented fissures (1).

**31.** **Frontal participation in rim of external naris:** does not participate (0); participates (1).

**32.** **Frontal, posterolateral process:** present (0); absent (1); inapplicable, premaxilla contacts parietal (?).

**33.** **Frontal participation in orbital margin:** participates (0); does not participate, excluded by prefrontal-postfrontal contact (1).

**34.** **Lacrimal:** absent, maxilla participates in orbit margin (0); present, maxilla excluded from orbit margin (1).

**35.** **Prefrontal participation in rim of external naris:** does not participate (0); participates (1).

**36.** **Postfrontal participation in orbital margin:** participates (0); does not participate, excluded by postorbital-frontal contact (1).

**37.** **Jugal participation in orbital margin:** participates (0); does not participate, excluded by maxilla-postorbital contact (1).

**38.** **Jugal, size and anteroposterior length:** large, with horizontal long axis, extends anteriorly at least one-third of orbital length (0); short, terminates around posterior orbital margin (1); very reduced and anteroposteriorly short with vertical long axis (2).

**39.** **Jugal, shape of anterior margin:** tapering, embayed by orbit margin, or contacts ‘lacrimal’ (0); squared off (1).

**40.** **Jugal-squamosal contact:** absent (0); present (1).

**41.** **Jugal-squamosal contact morphology:** subvertical and interdigitating (0); subhorizontal for most of length, not interdigitating (1); inapplicable, contact absent (?).

**42.** **Postorbital-squamosal contact:** present, excluding jugal from the margin of the supratemporal fenestra (0); absent, and jugal enters margin of the temporal fenestra (1).

**43.** **Postorbital posterolateral process length:** long, extending posteriorly for at least two-thirds of the temporal fenestra length (0); prominent, but not elongate, extending approximately one-third of temporal fenestra length (1); short or absent (2).

**44.** **Pineal foramen:** present (0); absent (1).

**45.** **Pineal foramen, surrounding elements:** enclosed entirely within the parietals (0); contacts the frontals or premaxillae anteriorly (1); inapplicable, pineal foramen absent (?).

**46.** **Pineal foramen-location relative to postorbital bar:** level with postorbital bar (0); just posterior to postorbital bar (1).

**47.** **Morphology of pineal foramen:** suboval (0); anteroposteriorly elongate and slot-like (1); inapplicable, pineal foramen absent (?).

**48.** **Inter-squamosal suture along the dorsal midline in lateral view:** low and rounded (0); raised ~1/3 orbit height dorsally relative to skull table (1); raised abruptly and substantially dorsally relative to skull table (2).

**49.** **Parietal vault in dorsal view:** mediolaterally narrow, lateral surfaces weakly concave or slightly sinuous (0); expanded to approximately one-third the mediolateral width of the skull, lateral surfaces convex, forming abrupt ‘lateral angle’ of Smith & Dyke (2008) (1); strongly expanded, equal to at least half the transverse width of the posterior cranium, lateral surfaces concave (2).

**50.** **Parietal, sagittal crest height:** crest absent, dorsal surface of parietal broad and flat (0); low, transversely convex (1); high, transversely compressed sheet (2); very high, forming convex dome in lateral view rising above the skull table (3).

**51.** **Parietal ornamentation adjacent to the pineal foramen:** ornamentation absent (0); ornamented with numerous anteroposteriorly oriented ridges that extend from the pineal foramen, surface flat or slightly concave along midline (1); parietal with raised midline ridge (2); ‘parietal table’ [triangular depression between pineal foramen and sagittal crest; Druckenmiller & Russell, 2008*b*] present (3).

**52.** **Parietal, anterior extension:** short or absent, parietal extends to the level of the temporal bar (0); long, parietal extends to orbital midlength or more anteriorly (1); very long, parietal extends to anterior orbit margin or more anteriorly (2).

**53.** **Squamosal arch, posterior margin in dorsal view:** dorsal processes extend anterolaterally (0); approximately straight, squamosal dorsal processes extend laterally from midline contact (1); V-shaped, squamosal dorsal processes extend posterolaterally (2).

**54.** **Squamosal arch, cross section of dorsal process of squamosal:** dorsoventral/mediolateral width subequal to or less than anteroposterior width (0); anteroposteriorly compressed (1).

**55.** **Temporal emargination:** moderately embayed, temporal bar arches dorsal to a horizontal line drawn through the tooth row (0); weakly embayed, or not embayed, temporal bar does not significantly arch dorsally (1).

**56.** **Temporal bar, dorsoventral thickness:** low, significantly less than height of orbit (0); high, subequal to 2/3 or greater than height of orbit (1).

**57.** **Squamosal, anterior extent:** ventral to postorbital bar (0); significantly posterior to postorbital bar (1).

**58.** **Inter-squamosal suture along the posterodorsal midline:** flat (0); prominent, ‘bulb-like’ posterior extension (1); low, mediolaterally broad posterior convexity in dorsal view (2).

**59.** **Squamosal-quadrate foramen:** absent (0); present (1).

**60.** **Squamosal-quadrate contact, length of ventromedial process of the squamosal:** short, approximately half the dorsoventral length of the quadrate shaft or less (0); long, extends further ventrally than half the quadrate shaft length (1).

**61.** **Squamosal, outline of posterior margin in lateral view:** approximately straight (0); dorsal portion inflected abruptly anterodorsally (1).

**62.** **Position of tooth row in lateral view:** collinear with the mandibular glenoid fossa (0); considerably higher than the glenoid fossa (1).

**63.** **Notochordal pit on occipital condyle:** absent (0); present (1); occipital condyle scored by multiple pits and deep grooves (2).

**64.** **Notochordal pit on occipital condyle, location:** centrally or at least partly within ventral two-thirds of condyle (0); comfortably within dorsal one-quarter of condyle (1); inapplicable, notochordal pit absent (?).

**65.** **Occipital condyle constriction:** complete, exoccipital facets are separated from the occipital condyle by a groove (0); incomplete because exoccipital facets contact the occipital condyle (1); or constricting groove altogether absent, even ventrally (2).

**66.** **Ventral process of the basioccipital:** absent, weakly developed or wide, flat, relatively smooth, with a thin plate present [small ‘step’ between condyle and ventral surface of basioccipital] (0); very prominent, ventrally projecting plate present (1).

**67.** **Foramen magnum, proportion of foramen enclosed by supraoccipital:** less than one-third (0); approximately half (1).

**68.** **Exoccipital-opisthotic body, dorsoventral height:anteroposterior width ratio:** <1.1 (0); 1.2–1.3 (1); >1.35 (2).

**69.** **Exoccipital, foramina in lateral surface:** one (0); two (1); three/four (2).

**70.** **Opisthotic, paraoccipital process length relative to height of exoccipital body:** subequal (0); long, at least 1.3 times as long as body height (1).

**71.** **Opisthotic, orientation of paraoccipital process relative to ventral surface of exoccipital in posterior view:** inclined dorsally (0); paraoccipital process oriented parallel to ventral surface of exoccipital (1); inclined ventrally (2).

**72.** **Opisthotic, morphology of articulation with suspensorium:** anterior surface of expanded lateral end makes broad contact with suspensorium (0); lateral end unexpanded, lateral/terminal surface makes narrow contact with suspensorium (1).

**73.** **Opisthotic, shaft of paraoccipital process cross section:** subcircular, dorsoventral height subequal to anteroposterior width (0); dorsoventrally flattened; anteroposterior width much greater than dorsoventral height (1).

**74.** **Opisthotic, shaft curvature seen in posteromedial view:** absent, shaft approximately straight (0); curves dorsodistally (1).

**75.** **Prootic, anteroventral process:** long, meaning that ventral anteroposterior length is much greater than dorsal anteroposterior length (0); short, dorsal anteroposterior length is slightly greater than ventral (1).

**76.** **Supraoccipital morphology in lateral view:** wider than tall (0); or taller than wide (1).

**77.** **Posteromedian ridge of supraoccipital:** present (0); absent (1).

**78.** **Posteromedian process of supraoccipital:** present (0); absent (1).

**79.** **Supraoccipital, minimum mediolateral width of exoccipital rami in posterior view:** a single ramus is substantially narrower than the foramen magnum (0); subequal to foramen magnum (1).

**80.** **Basisphenoid (or parabasisphenoid) contribution to the basioccipital tuberosities:** contributes, enclosing posterior half of tuber and forming part of the articular surface for the pterygoids (0); does not contribute (1).

**81.** **Deep notch in the posterior margin of the body of the basisphenoid [‘clivus’]:** absent (0); present (1).

**82.** **Basisphenoid-basioccipital connection in ventral view:** fontanelle absent (0); fontanelle present (1).

**83.** **Parasphenoid (or parabasisphenoid), morphology of ventral surface within interpterygoid vacuity:** mediolaterally concave (0); flat or weakly convex (1); bears distinct midline keel (2); inapplicable, parasphenoid does not extend far into posterior interpterygoid vacuity (?).

**84.** **Parasphenoid, posterior extent on midline:** terminates within anterior one-third of interpterygoid vacuity or more anteriorly (0); terminates just anterior to basioccipital-basisphenoid contact on ventral surface of basicranium (1); ventrally underlaps basioccipital so basisphenoid-basioccipital contact is not visible in ventral view (2); as state ‘2’ but also underlaps pterygoids ventrally (3).

**85.** **Parasphenoid, cultriform process length:** extremely short, effectively absent (0); present forming prominent anterior projection (1).

**86.** **Parasphenoid, ventral surface anteriorly:** covered by pterygoids anterior to the posterior interpterygoid vacuities (0); visible through V-shaped notch in posterior pterygoid contact anterior to posterior interpterygoid vacuities (1).

**87.** **Suborbital fenestra bordered by ectopterygoid and maxilla:** absent (0); present (1).

**88.** **Lateral palatal fenestration bordered by palatine and pterygoid:** absent (0); present (1).

**89.** **Element demarcating the anterior margin of the subtemporal fenestra:** the ectopterygoid and pterygoid together (0); exclusively the ectopterygoid (1).

**90.** **Palate, foramina between maxilla and vomer anterior to internal naris:** absent (0); present (1).

**91.** **Posterior extent of the vomers:** extend to the internal nares (0); extend posterior to the internal nares (1).

**92.** **Pterygoid-vomer contact:** pterygoid does not separate vomers along midline (0); pterygoid separates vomers along the midline posteriorly (1).

**93.** **Palatine, participation in the rim of the internal naris seen in ventral view:** participates (0); does not participate (1).

**94.** **Palatines, median contact:** do not contact (0); contact (1).

**95**. **Pterygoid, anterior termination:** tapering (0); transversely broad and interdigitates with vomer (1).

**96.** **Pterygoids, anterior interpterygoid vacuity:** absent (0); present (1).

**97.** **Pterygoids, anterior interpterygoid vacuity, mediolateral width:** narrow, approximately one-fifth of combined pterygoid width at vacuity midlength (0); broad, at least one-third of combined pterygoid width at vacuity midlength (1); inapplicable, vacuity absent (?).

**98.** **Pterygoids, posterior contact with basicranium:** loose, overlapping contact (0); firm sutural contact, ventral surface of pterygoids level with ventral surface of basioccipital (1); narrow anteromedial process of the posterior pterygoid contacts parabasisphenoid primarily or only, forming a butt joint (2).

**99.** **Pterygoids, midline contact posterior to posterior interpterygoid vacuity:** absent (0); present posteriorly, but very small (1); present, pterygoid contact for more than two-thirds of their anteroposterior length posterior to posterior interpterygoid vacuity (2).

**100. Pterygoid lateral to the posterior interpterygoid vacuities:** flat (0); forms ventrolaterally directed flange with long axis oriented posteromedially (1); relatively broad mediolaterally and forms anteroposteriorly oriented trough or dished, with a marked central depression (2).

**101.** **Pterygoid flange, posterior midline contact:** flanges do not contact on midline posterior to posterior interpterygoid vacuity (0); flanges contact on midline, enclosing semicircular fossa posterior to posterior interpterygoid vacuity (1); inapplicable, pterygoid flanges absent (?).

**102.** **Pterygoid, posterolateral portion of pterygoid:** does not form squared lappet (0); forms squared lappet that distinctly underlaps quadrate ramus of pterygoid (1).

**103.** **Posterior interpterygoid vacuities, ratio of maximum length to combined width:** <1.2 (0); 1.3–1.6 (1); 1.8–2.5 (2); >2.6 (3).

**104.** **Posterior interpterygoid vacuities, location of midpoint relative to anterior margin of subtemporal fossa:** posterior to anterior margin of fossa (0); approximately level with anterior margin of fossa or more anterior (1).

**105.** **Basioccipital body, exposure posterior to pterygoid midline contact:** absent, pterygoids cover ventral surface of basioccipital anterior to condyle (0); present, semioval portion of basioccipital exposed between pterygoids anterior to condyle (1).

**106.** **Posterior border of anterior interpterygoid vacuity:** bordered by pterygoid (0); bordered by parasphenoid (1); inapplicable, anterior interpterygoid vacuity absent (?).

**107.** **Anterior border of anterior interpterygoid vacuity:** bordered by pterygoid (0); bordered by vomer (1); inapplicable, anterior interpterygoid vacuity absent (?).

**108.** **Morphology of the posterior border of the anterior interpterygoid vacuity:** concave/rounded (0); parasphenoid projects into anterior interpterygoid vacuity (1); inapplicable, parasphenoid does not contact anterior interpterygoid vacuity, which is thus enclosed posteriorly by the pterygoids (?).

**109.** **Ectopterygoid/pterygoid boss/flange:** absent (0); ventrally deflected posterior margin forms flange (1); rugose ventral boss present (2).

**110.** **Ectopterygoid/pterygoid boss, transverse width:** approximately as wide mediolaterally as long anteroposteriorly (0); >1.5 times as wide mediolaterally as long anteroposteriorly (1).

**111.** **Shape of the mandible seen in dorsal/ventral view:** bowed medially anterior to glenoid (0); not significantly bowed (1).

**112.** **Mandible, symphysis length as measured by the number of alveoli adjacent to the symphysis [relative to the number of maxillary teeth or an estimate thereof]:** long symphysis, number of alveoli adjacent to symphysis equals *c*.0.4–0.5 of maxillary alveolar count (0); intermediate, ~0.20–0.30 (1); very short, only 1–2 alveoli adjacent to symphysis (2).

**113.** **Shape of the mandibular symphysis in ventral view:** tapers anteriorly (0); laterally expanded (1).

**114.** **Structure of the dentary along the ventral surface of the mandibular symphysis:** no ventral elaboration (0); forms raised ventral platform or sharp keel/ridge adjacent to symphysis (1).

**115.** **Contributions to the coronoid eminence laterally:** surangular mainly (0); dentary mainly (1).

**116.** **Length of retroarticular process:** shorter than or subequal to glenoid anteroposterior length (0); longer than glenoid (1).

**117.** **Orientation of glenoid:** articular surface faces dorsally or only slightly dorsomedially (0); strongly inclined dorsomedially (1).

**118.** **Mandible, posterior opening of Meckelian canal on medial surface [anterior margin of the adductor fossa]:** dorsoventrally low with V-shaped outline in medial view [anterior margin of adductor fossa 'poorly defined'] (0); dorsoventrally tall [anterior margin of adductor fossa 'well-defined'], occupying at least half the height of the mandible with curving outline in medial view (1).

**119.** **Mandible, prearticular/splenial-angular contact perforated by anteroposteriorly elongate, oval foramen [lingual mandibular fenestra]:** present, emarginates the medial wall of the angular dorsally (0); absent (1).

**120.** **Rounded medial flange formed by articular and prearticular anterior to the glenoid fossa in dorsal view:** present (0); absent (1); absent but anterior part of outline of glenoid in ventral view appears ‘squared-off’ (2).

**121.** **Mandible, prominent longitudinal trough occupies much of the lateral surface anterior to the glenoid [dentary, angular, surangular]:** absent (0); present, bounded ventrally by robust longitudinal, ventrolateral ridge (1).

**122.** **Mandible, retroarticular process, dorsoventral orientation of long axis:** posterodorsal (0); posteroventral or subhorizontal (1).

**123.** **Mandible, retroarticular process, mediolateral orientation of long axis:** directly posterior, in line with ‘anteroposterior’ long axis of glenoid (0); inflected slightly posteromedially (1).

**124.** **Mandible, dorsal rim of 'lingual mandibular fenestra' formed by:** prearticular (0); splenial (1); at prearticular-splenial contact (2); inapplicable, lingual mandibular fenestra absent (?).

**125.** **Splenial participation in mandibular symphysis:** does not participate (0); participates (1).

**126.** **Angular relative length and participation in mandibular symphysis:** short, extends less than half mandibular length (0); long, extending more than half mandibular length, but does not participate in the symphysis (1); very long, participates in symphysis (2).

**127.** **Surangular, fossa and longitudinal crest on medial surface anterior to glenoid:** prominent longitudinal crest forms ventral margin of deep, dorsomedially facing surangular fossa (0); prominent longitudinal crest forms medial margin of mediolaterally expanded dorsal surface of surangular bearing shallow, dorsally facing fossa (1); crest and surangular fossa weak or absent, dorsal portion of surangular 'blade-like' (2); dorsolaterally facing fossa bounded laterally by a sharp crest (3).

**128.** **Coronoid length and morphology:** long, approaching or participating in symphysis (0); small, superficial element that is often disarticulated and thus not preserved, but represented by a facet on the surangular (1).

**129.** **Prearticular, large dorsomedian trough or rugosity:** absent or weak (0); present (1).

**130.** **Articular, deep anteroposteriorly oriented cleft [notch] posterior to glenoid:** absent (0); present (1); cleft absent, but dorsal surface is strongly concave mediolaterally (2).

**131.** **Number of premaxillary teeth:** four (0); five (1); six (2); seven or more (3).

**132.** **Regularity of posterior premaxillary dentition:** homodont, distalmost alveolus similar size to more mesial alveoli (0); heterodont, reduced distalmost alveolus (1).

**133.** **Regularity of maxillary dentition:** homodont (0); heterodont (1).

**134.** **Diastema at premaxillary-maxillary suture:** absent (0); present (1).

**135.** **Spacing between mesial alveoli:** narrow, less than mesiodistal length of a single alveolus (0); wide, more than half, or greater than mesiodistal length of a single alveolus and compact bone divides premaxillary and a small number of mesial dentary alveoli (1); one-third of dentary and more than one half of upper tooth row (2).

**136.** **Enamel ‘striations’ (grooves):** present (0); absent (1).

**137.** **Form of apicobasally extending enamel ridges:** coarse (0); fine (1); absent (2).

**138.** **Number of maxillary teeth:** 12–19 (0); 20–27 (1); ≥28 (2).

**139.** **Cross-sectional shape of teeth in anterior half of tooth row:** round or sub-rounded (0); sub-triangular [= trihedral] (1); intermediate between states 0 and 1, with a flattened labial surface, but this surface [is] not substantially expanded anteroposteriorly [ =  subtrihedral] (2); suboval (3).

**140.** **Premaxilla, diameter of first alveolus:** not significantly smaller than third alveolus (0); less than half the diameter of third alveolus (1).

**141.** **Relative neck length:** the neck is shorter [<0.8 times] (0); subequal to (1); or longer than [>1.2 times] trunk length (2).

**142.** **Axial rib articulation:** articulates solely with the axis centrum (0); articulates partly with the atlas centrum (1).

**143.** **Axial rib facet morphology:** double-headed (0); single-headed (1).

**144.** **Atlas-axis complex, atlantal centrum [odontoid], participation in anterior rim of atlantal cup:** does not participate, excluded by atlantal neural arch–atlantal intercentrum contact (0); participates (1).

**145.** **Atlas-axis complex, hypophyseal ridge:** absent or low bulge (0); present and prominent (1).

**146.** **Atlas-axis complex, hypophyseal ridge morphology:** longitudinally elongate ventral ridge of approximately equal prominence for its entire length (0); substantially more prominent anteriorly, forming an anteroventral eminence (1); located posteriorly (2).

**147.** **Atlas-axis complex, hypophyseal ridge location:** extends across both atlantal centrum, and axial centrum (0); does not contact the axial centrum (1); inapplicable, hypophyseal ridge absent (?).

**148.** **Atlas rib/rib facet or rib-like projection:** absent (0); rib present, contacts atlas *via* a distinct rib facet [sometimes co-ossified to atlas] (1); posteroventral projection resembling a fused atlantal rib, but lacking evidence of a rib facet (2).

**149.** **Axial intercentrum, size:** small, restricted to ventral surface of atlas-axis complex (0); large, wedge-shaped element that extends dorsally (1).

**150.** **Axial neural spine:** transversely narrow (0); transversely broad (1).

**151. Axial-atlas-axis complex, length:height ratio of centra:** <1.1 (0); 1.15-1.45 (1); >1.5 (2).

**152.** **Number of cervical vertebrae:** <15 (0); 18–23 (1); 24–29 (2); 30–36 (3); 37–49 (4); 50–59 (5); >60 (6).

**153.** **Proportions of anterior–middle cervical centra:** substantially shorter than high [length <0.7 x height] (0); approximately as long as high (1); substantially longer than high (2); even longer, corresponding to the ‘can’ shaped morphology of Otero et al. (2016a) or ‘elongate’ morphology of O’Keefe and Hiller (2006).

**154.** **Lateral surfaces of anterior cervical centra:** longitudinal ridge absent (0); present (1).

**155.** **Cervical centra, ventral notch:** absent, centra subcylindrical (0); present, centra 'dumbell’ or ‘binocular’ shaped (1).

**156.** **Cervical vertebrae, subcentral foramina and foramina on the dorsal surface of the centrum, within the neural canal:** both absent (0); both present (1); dorsal foramina present, but subcentral foramina very small or absent (2).

**157.** **Anterior cervical neural spines, morphology:** curve posterodorsally (0); inclined straight posterodorsally (1); inflected anterodorsally (2); inapplicable in some pistosaurians that have extremely low neural spines (?).

**158.** **Posterior cervical neural spines, morphology:** curve posterodorsally (0); inclined straight posterodorsally (1); inflected anterodorsally (2); inapplicable in some pistosaurians that have extremely low neural spines (?).

**159.** **Posterior cervical neural spines, height relative to centrum:** substantially shorter than centrum (0); subequal (1); substantially taller [equal to or greater than 1.2 times centrum height] (2).

**160.** **Rib facets of the anterior–middle cervical vertebrae:** rib facets broadly separated (0); two co-joined rib facets (1); mixture of single- and double-headed anterior cervical ribs (2); one rib facet (3).

**161.** **Rib facets of the posterior cervical vertebrae:** rib facets broadly separated (0); two co-joined rib facets (1); one rib facet (2).

**162.** **Cervical rib facets location:** ventrolaterally on centrum, do not contact neural arch peduncles (0); more dorsally, contact neural arch peduncles (1).

**163.** **Cervical ribs, size and orientation of distal processes:** marked anterior and posterior processes throughout cervical rib series, combined long axis of processes oriented approximately anteroposteriorly (0); processes reduced, especially anterior process, combined long axis oriented posteroventrally (1); large, anteroposteriorly expansive, sheet-like ribs with prominent processes (2).

**164.** **Cervical zygapophyses, combined width:** broader than the centrum (0); subequal to the centrum (1); or distinctly narrower than the centrum (2).

**165.** **Cervical centra, median ventral surface:** approximately flat or convex (0); bears a rounded midline ridge [*cf*. Tarlo, 1960] (1); or bears a sharp keel (2).

**166.** **Cervical centra, paired lateral ridges on ventral surface:** absent (0); present (1).

**167.** **Cervical zygapophyses, orientation:** horizontal (0); dorsomedially facing (1).

**168.** **Cervical zygapophyses, median contact between left and right zygapophyseal facets:** absent for most/all of length (0); present for most of anteroposterior length (1).

**169.** **Cervical zygapophyseal facets:** planar (0); transversely concave/convex (1).

**170.** **Dorsal and posterior cervical neural spines, dorsoventrally elongate groove on posterior surface:** absent (0); present (1).

**171.** **Cervical vertebrae, proportions of anterior cervical neural spines:** taller than their anteroposterior length (0); longer than tall (1); anteroposteriorly short and 'rod-like', approximately as long anteroposteriorly as the transverse width (2); as long as tall (3).

**172.** **Cervical vertebrae, shape of neurocentral suture in anterior–middle cervical vertebrae in lateral view:** rounded, ventrally convex (0); V-shaped (1); extends far ventrally so that neural arch contacts dorsal part of rib facet (2); extends far ventrally but is evenly convex and does not contact rib facet (3).

**173.** **Cervical centrum, proportional width:** mediolateral width subequal to height or less (0); at least 1.2 times as wide mediolaterally as high dorsoventrally (1).

**174.** **Cervical neural spines, apices of posteriormost cervical and anterior dorsal neural spines:** weakly expanded and convex (0); apex transversely expanded into prominent spine table (1).

**175.** **Anterior cervical centra, small, semi-oval ‘lip’ extends ventrally from anterior articular surface:** no (0); small, transversely narrow lip (1); broad, prominent lip, at least 0.5 times the width of the centrum (2).

**176.** **Posterior cervical rib facets:** face laterally (0); or posterolaterally (1).

**177.** **Middle­ to posterior dorsal transverse processes, distal articular facet:** dorsoventrally tall oval, perhaps composed of two weakly divided rib facets (0); composed of only a single subcircular facet (1).

**178.** **Height of dorsal neural spines in lateral view:** less than or equal to the height of the centrum (0); conspicuously taller than the centrum (1); more than twice as tall as the centrum (2).

**179.** **Number of dorsal vertebrae:** 17–19 (0); 20–23 (1); >24 (2).

**180.** **Number of pectoral vertebrae:** 2–4 (0); 5–7 (1).

**181.** **Dorsal neural arch height:** tall, base of transverse process located dorsal to midheight of neural canal (0); short, transverse process adjacent to neural canal (1).

**182.** **Dorsal transverse processes, orientation in middle dorsal region:** approximately horizontal [laterally oriented] (0); inclined significantly dorsolaterally (1).

**183.** **Dorsal neural spines, strong anteroposterior constriction at base:** absent (0); present (1).

**184.** **Dorsal neural spines, mediolateral width of apices in mid–posterior dorsal neural spines:** unexpanded, transversely narrow relative to anteroposterior width (0); mediolaterally thick, subequal to anteroposterior width (1); alternate spines expanded laterally to one side (2).

**185.** **Posteriormost dorsal rib facets:** prominent transverse process located entirely on neural arch (0); rib facet split between neural arch and centrum [‘sacralised’], but bears a typical posterior dorsal rib (1).

**186.** **Sacral ribs:** cylindrical and slightly expanded towards distal end (0); transversely expanded, dorsoventrally thin and sheet-like (1).

**187.** **Caudal vertebral count:** 25–30 (0); 33–35 (1); 36–40 (2); >40 (3).

**188.** **Caudal ribs facet location in proximal–middle caudal vertebrae:** located dorsally, contacting or almost contacting neural arch (0); placed dorsally but neural arch does not form part of facet (1); at midheight of centrum or lower (2).

**189.** **Caudal centra, outline of middle caudal centra in anterior view:** suboval (0); subrectangular, chevron facets widely spaced and located ventrolaterally, ventral surface approximately flat giving a subrectangular appearance to centrum in anterior view (1).

**190.** **Caudal centra, length:height ratio of proximal caudal centra:** >0.85 (0); 0.6-0.8 (1); <0.55 (2).

**191.** **Caudal centrum, subcentral foramina on ventral surface:** paired lateral foramina (0); single midline foramen (1).

**192.** **Caudal vertebrae, chevron facet:** located equally on anterior and posterior edges of the centrum (0) or mainly on the posterior edge, low, mound-like eminence may be present on ventrolateral surface of centrum anteriorly (1).

**193.** **Middle and distal caudal vertebrae, chevron facets:** flush with level of ventral surface of centrum (0); project significantly ventrally (1).

**194.** **Proximal caudal centra: width to height ratio:** 0.9-1.1 (0); >1.1 (1).

**195.** **Distalmost caudal vertebrae, forming ‘pygostyle’:** absent (0); present (1).

**196.** **Ratio of coracoid to scapular length:** > or equal to 1.9 (0); 1.6–1.9 (1) < or equal to 1.6 (2).

**197.** **Anteromedial margin of the coracoid:** does not contact the scapula (0); contacts the scapula (1).

**198.** **Anteromedial margins of the coracoids:** do not contact the dermal girdle elements [clavicle and interclavicle] (0); contact the dermal girdle elements (1).

**199.** **Scapula morphology:** dorsal blade expanding ventrally to form acetabular region, lacks expanded ventral plate (0); triradiate with expansive ventral plate (1).

**200.** **Contact of the ventral plates of the scapulae along the midline:** do not meet along the midline (0); meet along the ventral midline (1).

**201.** **Scapula blade, outline of anterior margin in lateral view:** approximately straight, weakly concave or weakly convex (0); pronounced posterodorsal inflection (1); distinct concave region anterodorsally (2).

**202.** **Shape of the anterolateral margin of the scapula where the dorsal ramus meets the ventral ramus:** flat or gently convex (0); forms prominent ridge or shelf (1); inapplicable, ventral plate not developed (?).

**203.** **Scapular blade, anteroposterior width at distal end:** subequal to width at midlength (0); narrow, tapering dorsally (1); broad, distal part expanded relative to midlength (2).

**204.** **Scapula blade, length relative to posterior process of scapula:** blade longer (0); blade subequal to or shorter than (1).

**205.** **Scapula blade, angle relative to ventral scapula margin:** vertical/subvertical [70-90 degrees] (0); intermediate posterodorsal inclination [45-60 degrees] (1); strong posterodorsal inclination [30-45 degrees] (2).

**206.** **Scapula blade, medial surface:** smoothly convex or flat (0); robust buttress oriented parallel to long axis of blade (1).

**207.** **Coracoid, posterolateral cornu:** does not extend as far laterally as glenoid (0); extends to level of glenoid (1); extends lateral to glenoid (2).

**208.** **Coracoid, median fenestra/large embayment:** absent, although intercoracoid contact may be slightly split posteriorly (0); median embayment present (1); posterior processes strongly divergent forming prominent V-shaped or otherwise mediolaterally narrow emargination (2).

**209.** **Coracoid, shape of anterior process:** anteroposteriorly long and transversely broad, approximately rectangular (0); anteroposteriorly long and transversely narrow (1); anteroposteriorly short and subtriangular (2).

**210.** **Coracoid, posterior margin, outline in dorsal view:** oriented approximately mediolaterally, may be convex, straight or weakly concave (0); anterolaterally oriented (1); possesses a distinct posterior process adjacent to midline (2); oriented posterolaterally (3).

**211.** **Coracoid plate, perforations/large foramina:** absent (0); present (1).

**212.** **Coracoid, dorsoventral height of anterior process:** dorsoventrally low and thus plate-like (0); taller dorsoventrally than mediolaterally (1).

**213.** **Coracoid, anterior process orientation:** extends approximately anteriorly (0); inflected anterolaterally (1).

**214.** **Coracoid, robust buttress on dorsal [visceral] surface connecting glenoid to median symphysis, orientation:** posteromedially (0); mediolaterally, and forms posterior margin of an anterior depression (1); mediolaterally oriented, but located anteriorly so anterior depression is absent (2).

**215.** **Coracoid, ventral projection/process extends from intercoracoid symphysis:** absent (0); present (1).

**216.** **Coracoid, low, mediolaterally oriented buttress connecting glenoid to median symphysis on ventral surface:** present (0); absent (1); mediolaterally oriented shelf or sharp crest extends anteriorly from coracoid surface bounding pectoral fenestra posteriorly (2).

**217.** **Clavicle/interclavicle complex, median fenestra:** absent (0): present (1).

**218.** **Contact of the clavicles along the midline:** present (0); absent (1).

**219.** **Clavicle/interclavicle complex, shape of anterior margin:** markedly concave, mediolateral width of concavity at least 1.25 times the anteroposterior depth (0); anteriorly convex or pointed (1); transversely broad and almost straight (2); small, transversely narrow, weakly concave region (3).

**220.** **Median pelvic bar:** present (0); absent (1).

**221.** **Ilium curvature shaft in lateral view:** appears straight, pelvic articular end equally expanded anteriorly and posteriorly (0); curves anterodorsally, articular end expanded only anteriorly (1); sigmoidal (2).

**222.** **Ilium, rotation of dorsal blade relative to long axis of proximal articular end:** ends perpendicular to one another (0); ends rotated by approximately 45 degrees relative to one another (1).

**223.** **Ilium, shape of dorsal expansion:** subequal anterior and posterior expansion, occupies dorsal half of ilium (0); subequal anterior and posterior expansion (or narrowing) confined to dorsal one-third of ilium (1); asymmetrical, extends much further posterodorsally than anteriorly, dorsal surface slopes posterodorsally (2); inapplicable, dorsal expansion absent (?).

**224.** **Ilium, anteroposterior width of dorsal expansion:** tapering, less than minimum shaft width (0); slight, just greater than minimum shaft width (1); expanded, between 1.5–2.0 times the minimum anteroposterior width of the shaft (2); large, over 2.5 times minimum shaft width (3).

**225.** **Ilium, tubercle on posterior surface around midlength:** absent (0); present as a tubercle (1); rugose, proximodistally oriented crest present (2).

**226.** **Ilium, cross section of shaft around midlength:** subcircular (0); mediolaterally compressed, suboval (1).

**227.** **Ilium ratio of length to minimum anteroposterior width:** <3.0 (0); 4.0–5.2 (1); >5.3 (2).

**228.** **Ilium, pronounced, broad fossa on medial surface of the dorsal end:** present (0); absent or weak (1).

**229.** **Pubis, ratio of anteroposterior length to minimum mediolateral width:** < or equal to 1.2 (0); >1.3 (1).

**230.** **Pubis, anterolateral cornu:** absent or weak and rounded, extending less far laterally than acetabulum (0); or present, extending further laterally than acetabulum (1).

**231.** **Ischium, length to width ratio:** < or equal to 0.9 (0); 1.0–1.3 (1); 1.4–1.8 (2); >1.85 (3).

**232.** **Limbs, postaxial accessory ossicles:** absent, or small, round elements appearing late in ontogeny without well-defined articular surfaces (0); present as small elements (1); present as large, well-defined elements contacting other limb bones (e.g. humerus, ulna) *via* well-defined articular surfaces, ossification of these elements is often late but their presence can be inferred by the presence of articular surfaces (2).

**233.** **Second postaxial accessory ossicle articulating with propodial:** absent (0); present (1).

**234.** **Limbs, preaxial accessory ossicles:** absent (0); present but small (1); present as large, well-defined elements contacting other limb bones (e.g. humerus, radius) *via* well-defined articular surfaces, ossification of these elements is often late but their presence can be inferred by the presence of articular surfaces (2).

**235.** **Forefin, ratio of proximodistal length excluding humerus to maximum anteroposterior width of humerus/proximal epipodials [aspect ratio]:** <3.0 (0); 3.1–3.5 (1); >3.6 (2).

**236.** **Ratio of forelimb length to trunk length:** <0.8 (0); >0.9 (1).

**237.** **Difference in the axes of propodial and rest of the paddle in the forelimb:** proximodistal axis of digits and tarsals/carpals collinear with propodial long axis (0); digits/tarsal/carpal axis extends posterodistally relative to propodial long axis because proximodistal length of radius/tibia is substantially greater than that of the ulna/fibula (1).

**238.** **Propodials, dorsal and ventral surfaces of distal half:** uniformly convex or flat with robust pre-and postaxial margins (0); weakly concave region separates central, convex portion from strongly tapering, flange-like pre- and postaxial margins (1).

**239.** **Humerus, long axis curvature in anterior view:** straight or almost straight (0);

**240.** **Femur, long axis curvature in anterior view:** straight or almost straight (0); pronounced dorsodistal curve (1); pronounced ventrodistal curve (2).

**241.** **Ratio of humerus to femur length:** <0.85 (0); 0.9–1.1(1); >1.1 (2).

**242.** **Epipodials, ratio of radius to tibia length:** <0.89 (0); 0.9–1.09(1); 1.1–1.3 (2); >1.4 (3).

**243.** **"Tongue-and-groove" articulation between propodial and epipodial:** absent, distal articular surfaces of propodials smooth (0); present, deep recesses in distal articular surfaces of propodials accommodate highly convex proximal surfaces of epipodials (1); absent, but a prominent anteroposteriorly oriented ridge bisects the epipodial facets (2).

**244.** **Humerus length *versus* width ratio:** >2.9 (0); 2.3–2.7 (1); 1.7–2.2 (2); < 1.6 (3).

**245.** **Preaxial margin of distal humerus in dorsal or ventral view:** straight or convex (0); concave [distal humerus expands anteriorly], but anterior expansion relatively small, substantially less than posterior expansion (1); concave, and anterior expansion is large, approaching the size of the posterior expansion (2).

**246.** **Sharp longitudinal ridge on anterior margin of humerus:** absent (0); present (1).

**247.** **Shape of the distal end of propodials:** uniformly convex (0); propodials distinctly angled for articulation with the epipodials (1).

**248.** **Humerus, angle between long axes of epipodial facets in dorsal view:** oblique (0); close to 180 degrees (1).

**249.** **Humerus, inclination of proximal end in dorsal view:** inclined posteriorly so that the proximal portion of the anterior margin is convex in dorsal view [often a low mound is located proximally on anterior surface] (0); not inclined, extends proximally so shaft appears straight (1); inclined anteriorly so shaft appears sigmoidal (2).

**250.** **Humerus, shallow groove on ventral surface between epipodial facets (flexor groove):** present and prominent (0); present but anteroposteriorly short and shallow (1); absent (2).

**251.** **Femoral length *versus* width ratio:** >2.8 (0); 2.1–2.7 (1); 1.55–2.0 (2); < 1.5 (3).

**252.** **Humerus, tuberosity morphology:** narrow and projects dorsally (0); broad and projects posterodorsally [tilted] (1).

**253.** **Femur, trochanter morphology:** narrow and projects dorsally (0); broad and projects slightly posterodorsally [slightly tilted] (1).

**254.** **Ratio of radius length to maximum width:** >2.7 (0); 1.1–1.5 (1); 0.8–1.0 (2); < or equal to 0.75 (3).

**255.** **Ratio of tibia length to maximum width:** >2.5 (0); 1.1–1.8 (1); 0.8–1.0 (2); < or equal to 0.75 (3).

**256.** **Radius morphology:** preaxial margin concave (0); straight or convex (1).

**257.** **Radius, prominent anterior flange extends from anteroproximal surface:** absent (0); present (1).

**258.** **Ulna morphology:** postaxial margin concave (0); convex (1).

**259.** **Tibia morphology:** preaxial side of tibia concave (0); convex or straight (1).

**260.** **Ulna, expansion of distal end relative to shaft:** absent or very weak (0); present (1).

**261.** **Epipodial foramen [=spatium interosseum; =antebrachial foramen]:** present, proximodistal length slightly shorter than epipodials (0); present but short, proximodistal length less than half epipodial length (1); absent (2).

**262.** **Ratio of maximum radius length to maximum ulna length:** 1.0–1.3 (0); 1.4–1.7 (1); > or equal to 2.0 (2).

**263.** **Radius, posterodistal facet for intermedium:** absent (0); present (1).

**264.** **Tibia, posterodistal facet for intermedium:** absent (0); present (1).

**265.** **Width of epipodials of the hindlimb:** tibia larger (0); widths within 10% of each other (1); fibula larger (2).

**266.** **Anterior (I) and central (II) distal tarsals/carpals:** offset relative to proximal tarsals/carpals so distal elements articulate with multiple proximal elements (0); in line with proximal tarsals/carpals, lacking anteroproximal or posteroproximal articular surfaces (1).

**267.** **Interlocking distal phalanges:** absent, digits splayed (0); present, digits parallel (1).

**268.** **Position of the fifth metapodial:** lies in the metapodial row (0); shifted proximally so that the proximal half is in the distal mesopodial row (1); shifted proximally so the entire fifth metapodial is in the mesopodial row in manus (2).

**269.** **Metapodials, morphology of proximal ends:** all metapodials form straight, anteroposteriorly oriented butt contacts with distal tarsals (0); at least one metapodial possesses a bifaceted proximal articular surface (1).

**270.** **Phalanx proportions:** long and slender [~2-3 times as long proximodistally as broad anteroposteriorly] (0); short and robust (1).
