# Supplementary material for: The osteology, taxonomy, and phylogenetic affinities of the Early Jurassic plesiosaur Lusonectes sauvagei
Source: PeerJ. 2026 Feb 2;14:e20611. doi: 10.7717/peerj.20611 (PMC12875220; doi:10.7717/peerj.20611)

**Supplementary Information IV**

**The osteology, taxonomy, and phylogenetic affinities of the Early Jurassic plesiosaur *Lusonectes sauvagei***

Sven Sachs and Daniel Madzia

**Figure S1.** Parsimony analysis using equal weights, utilizing the ‘conservative’ operational taxonomic unit of *Lusonectes sauvagei*. Strict consensus tree. Numbers on nodes show Bremer support values.


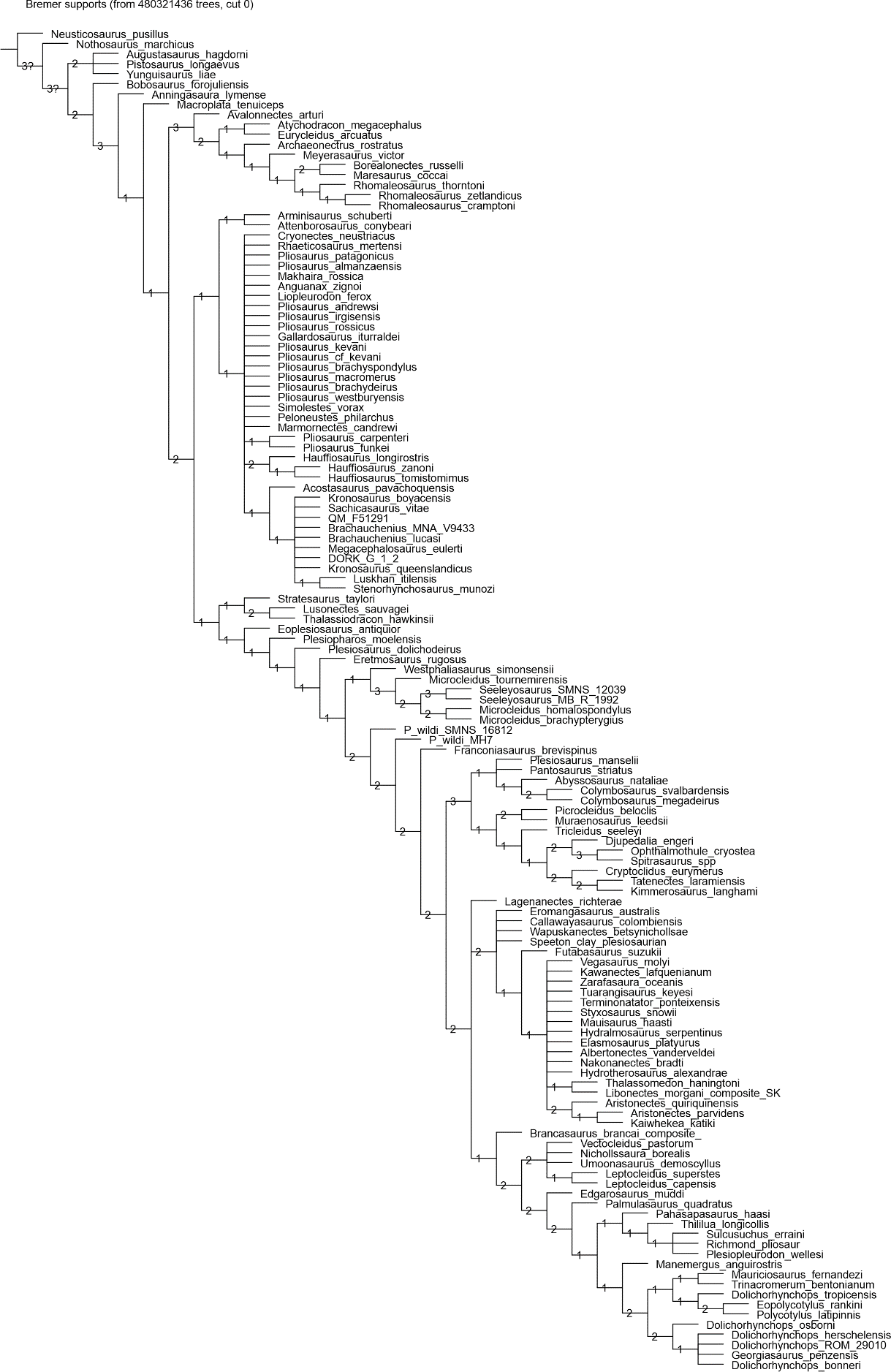


**Figure S2.** Parsimony analysis with implied weighting (*K* = 9), utilizing the ‘conservative’ operational taxonomic unit of *Lusonectes sauvagei*. Strict consensus tree.


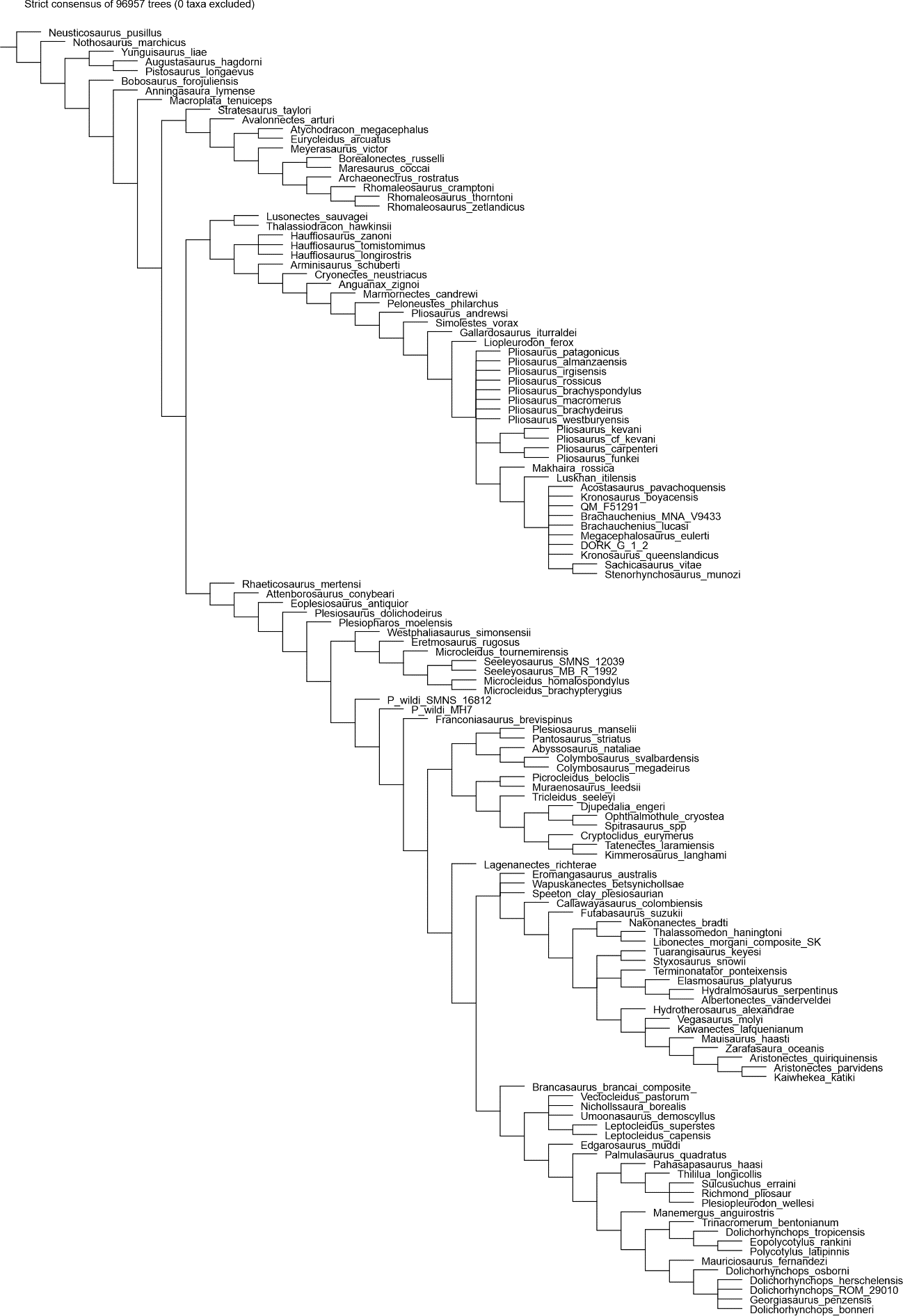


**Figure S3.** Parsimony analysis with implied weighting (*K* = 9), utilizing the ‘conservative’ operational taxonomic unit of *Lusonectes sauvagei*. Symmetric Resampling.


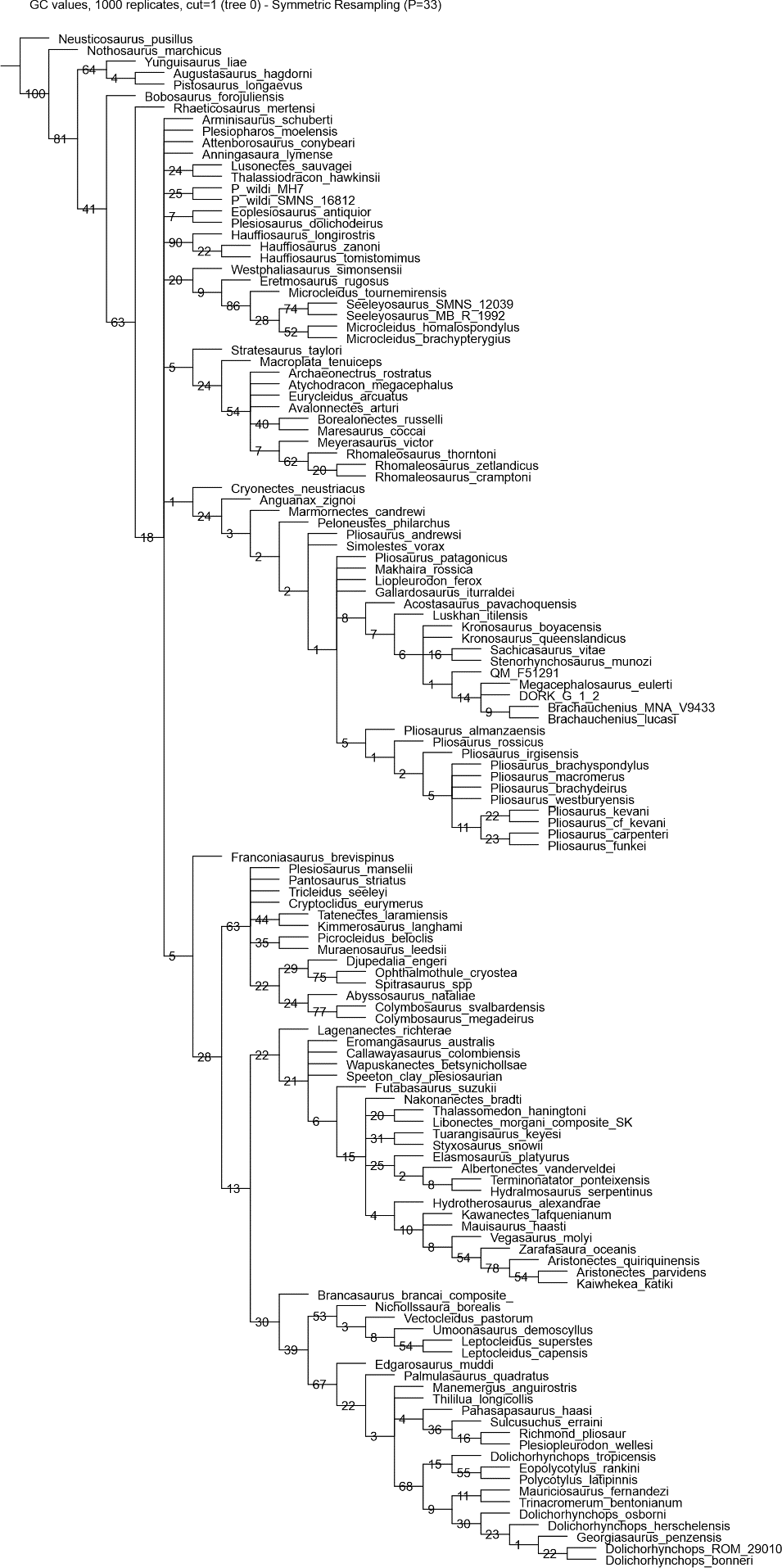


**Figure S4.** Parsimony analysis with implied weighting (*K* = 15), utilizing the ‘conservative’ operational taxonomic unit of *Lusonectes sauvagei*. Strict consensus tree.


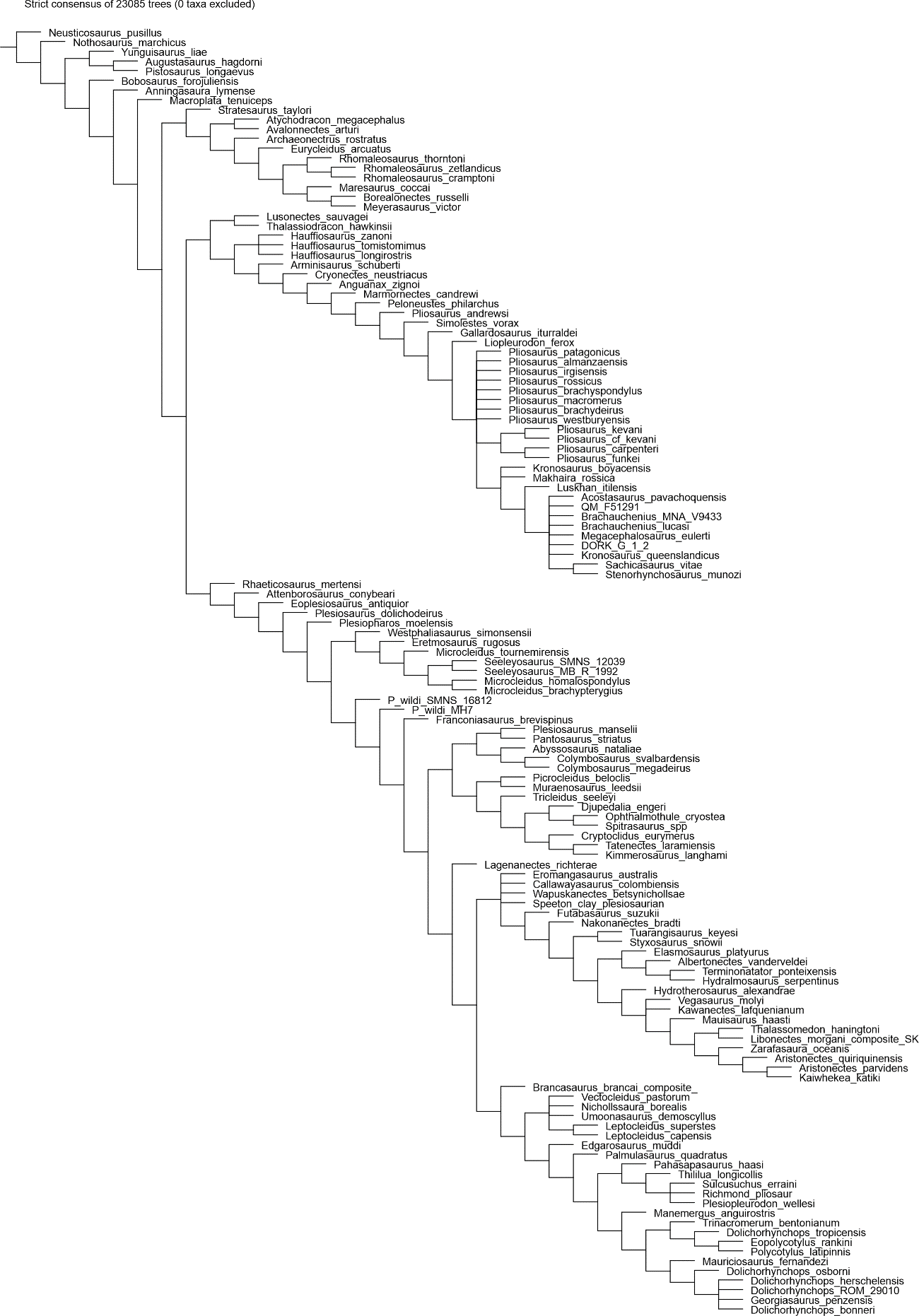


**Figure S5.** Parsimony analysis with implied weighting (*K* = 15), utilizing the ‘conservative’ operational taxonomic unit of *Lusonectes sauvagei*. Symmetric Resampling.


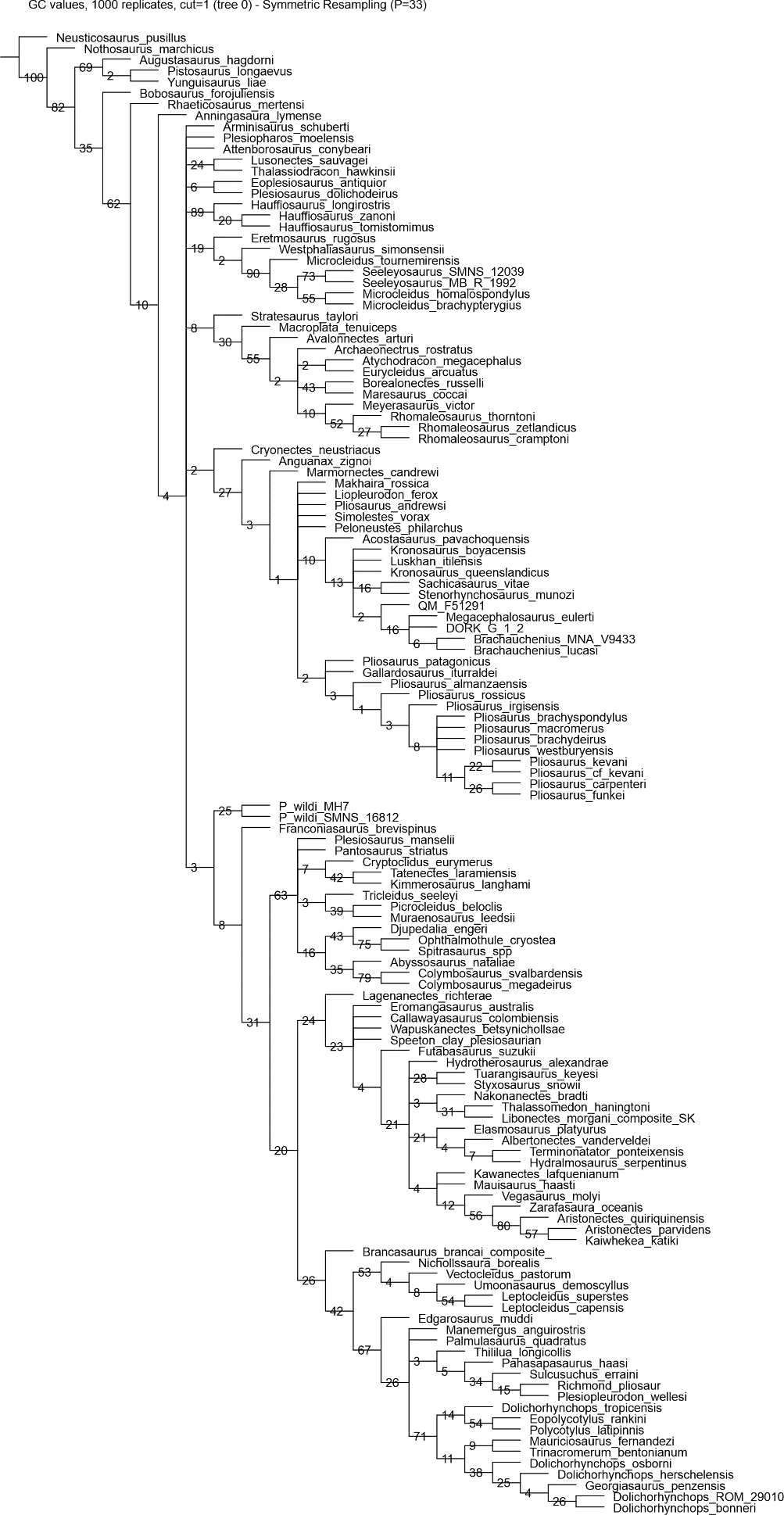


**Figure S6.** Parsimony analysis using equal weights, utilizing the ‘experimental’ operational taxonomic unit of *Lusonectes sauvagei*. Strict consensus tree. Numbers on nodes show Bremer support values.


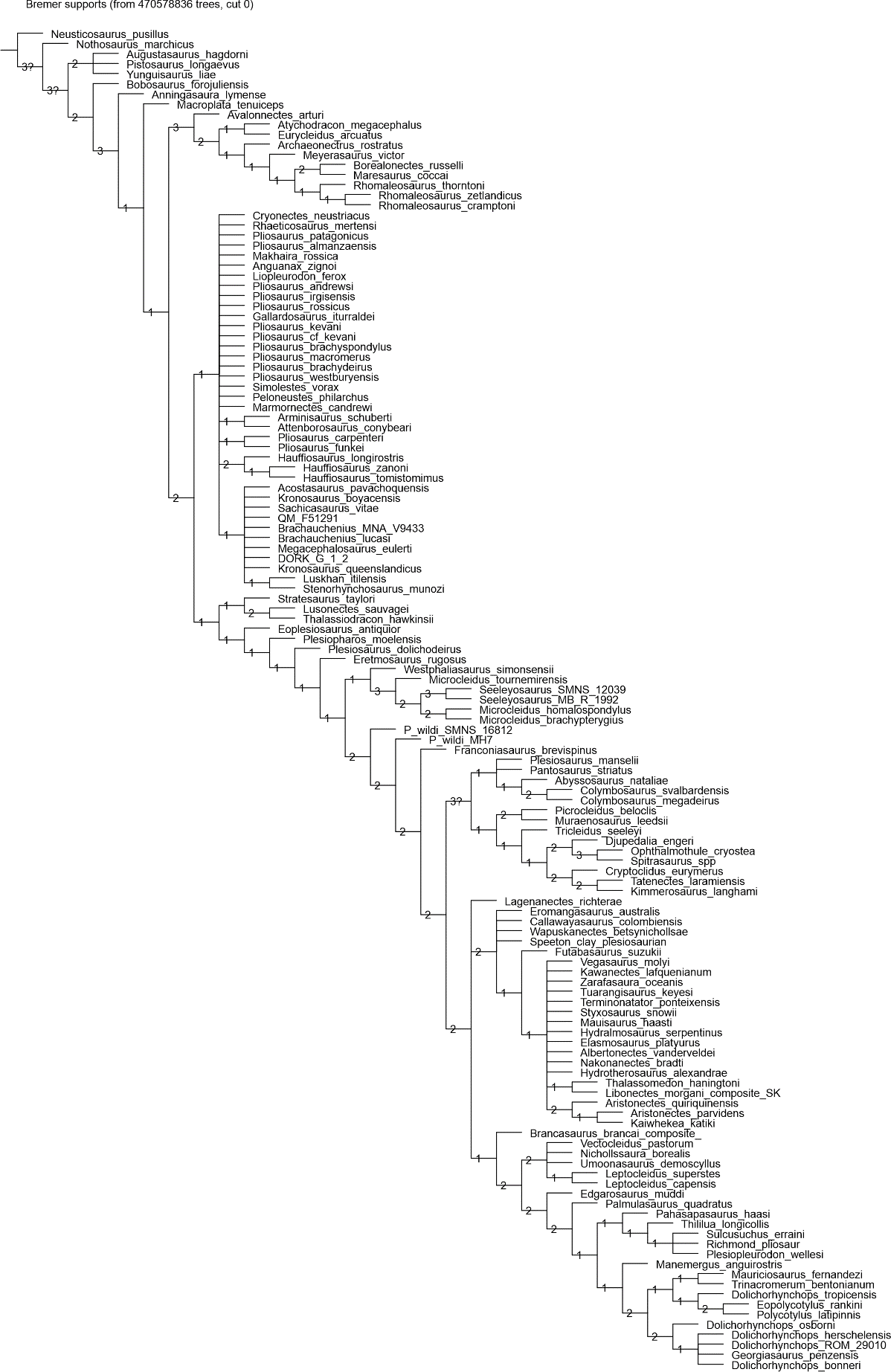


**Figure S7.** Parsimony analysis with implied weighting (*K* = 9), utilizing the ‘experimental’ operational taxonomic unit of *Lusonectes sauvagei*. Strict consensus tree.


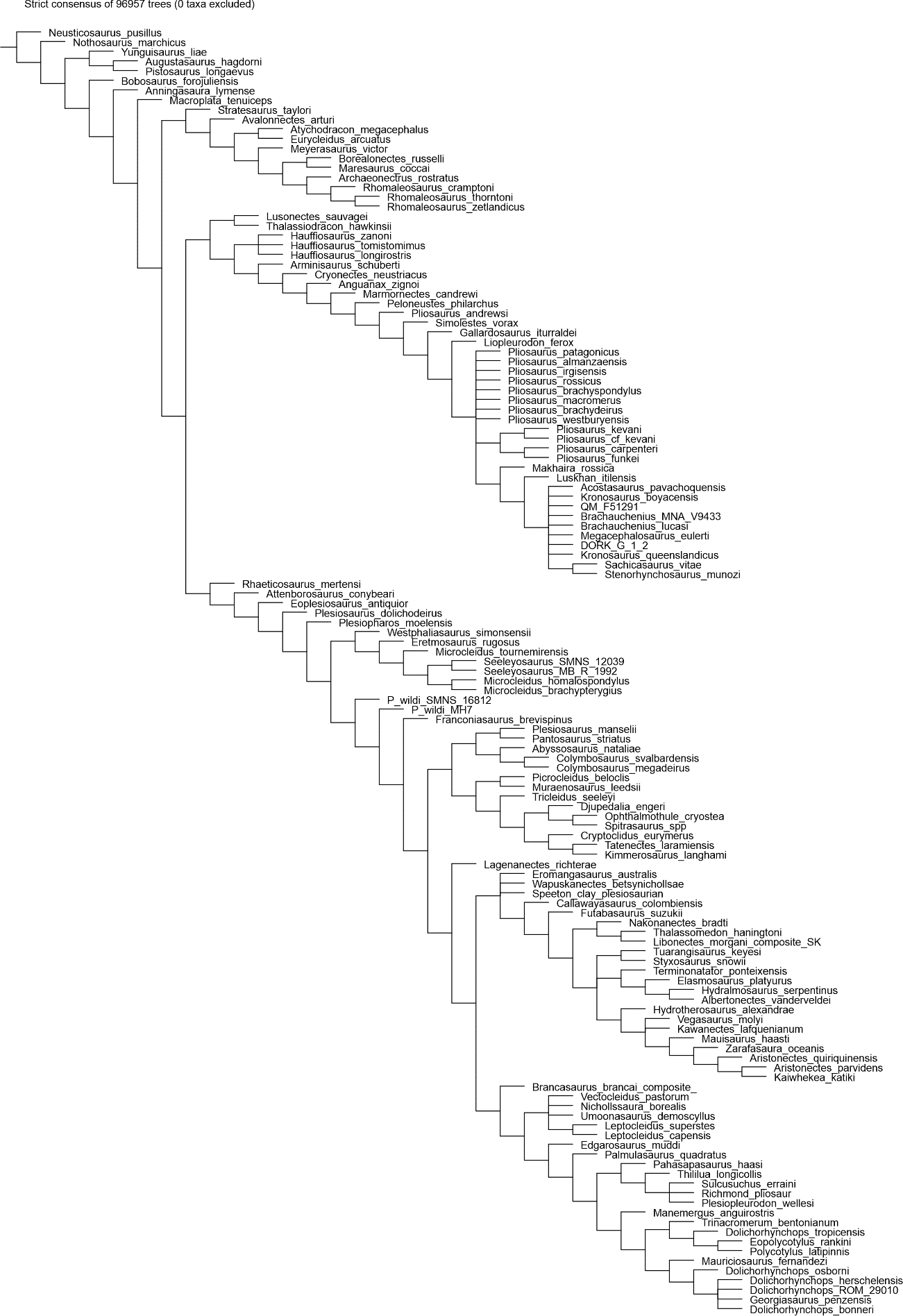


**Figure S8.** Parsimony analysis with implied weighting (*K* = 9), utilizing the ‘experimental’ operational taxonomic unit of *Lusonectes sauvagei*. Symmetric Resampling.


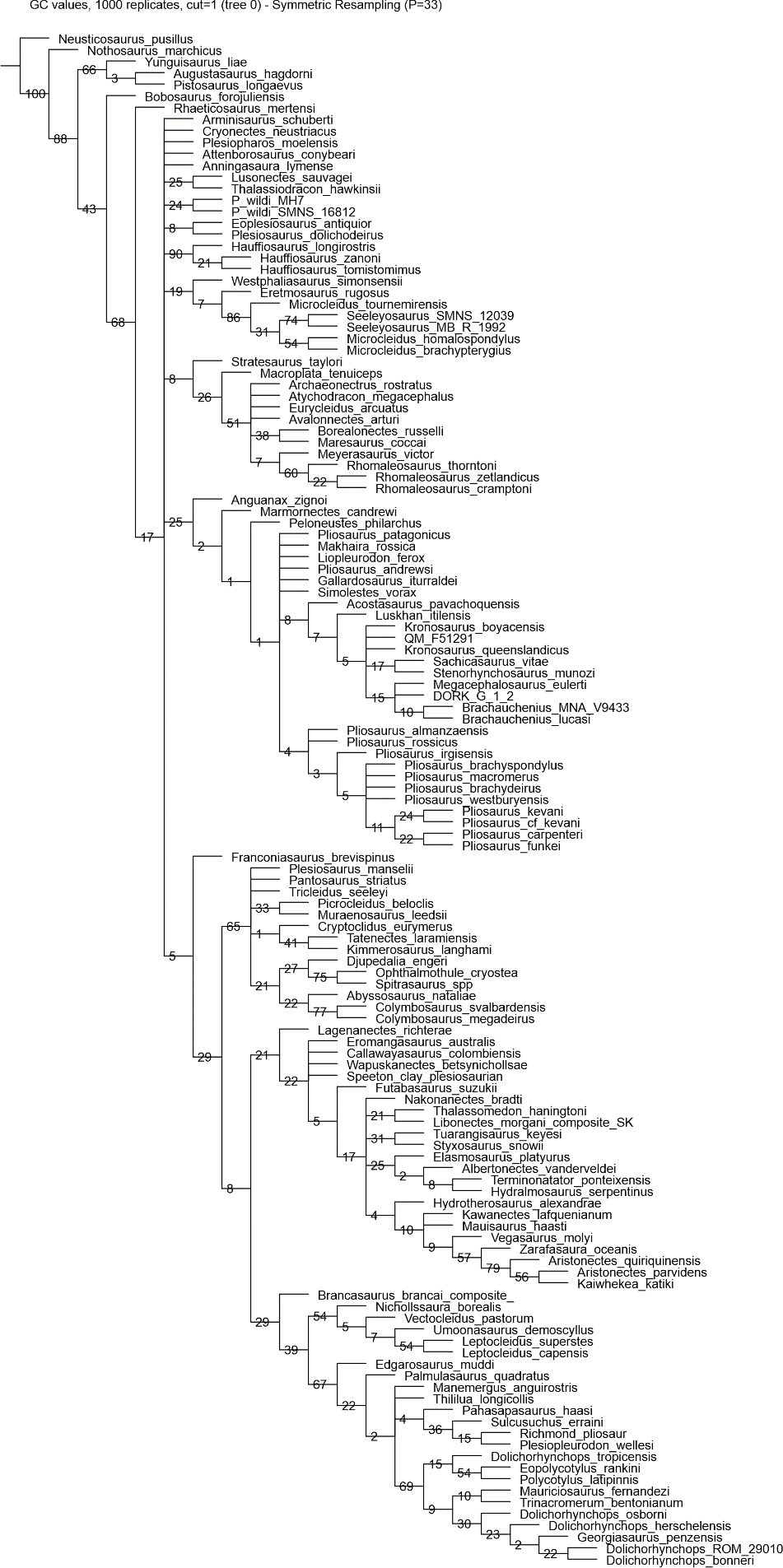


**Figure S9.** Parsimony analysis with implied weighting (*K* = 15), utilizing the ‘experimental’ operational taxonomic unit of *Lusonectes sauvagei*. Strict consensus tree.


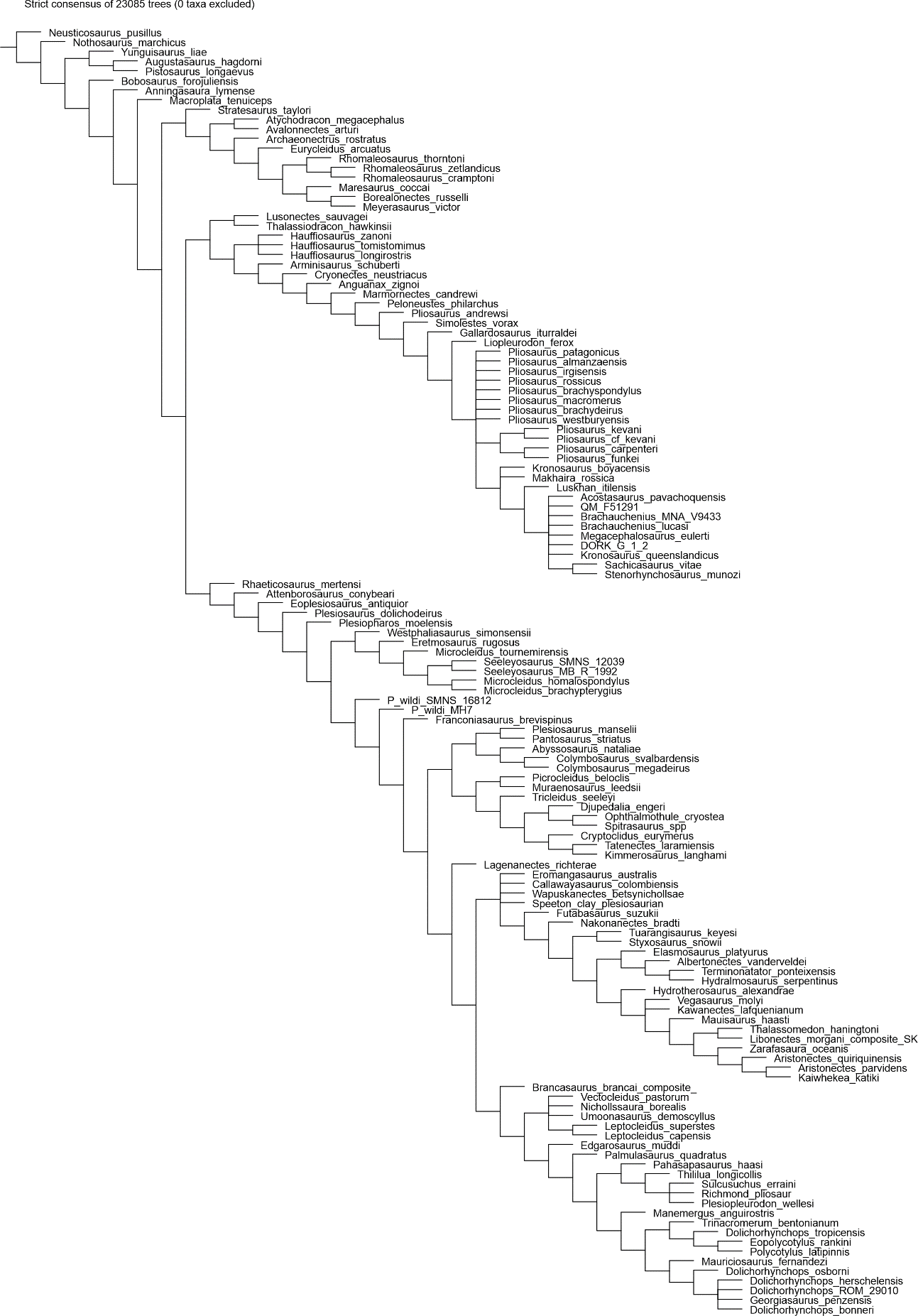


**Figure S10.** Parsimony analysis with implied weighting (*K* = 15), utilizing the ‘experimental’ operational taxonomic unit of *Lusonectes sauvagei*. Symmetric Resampling.


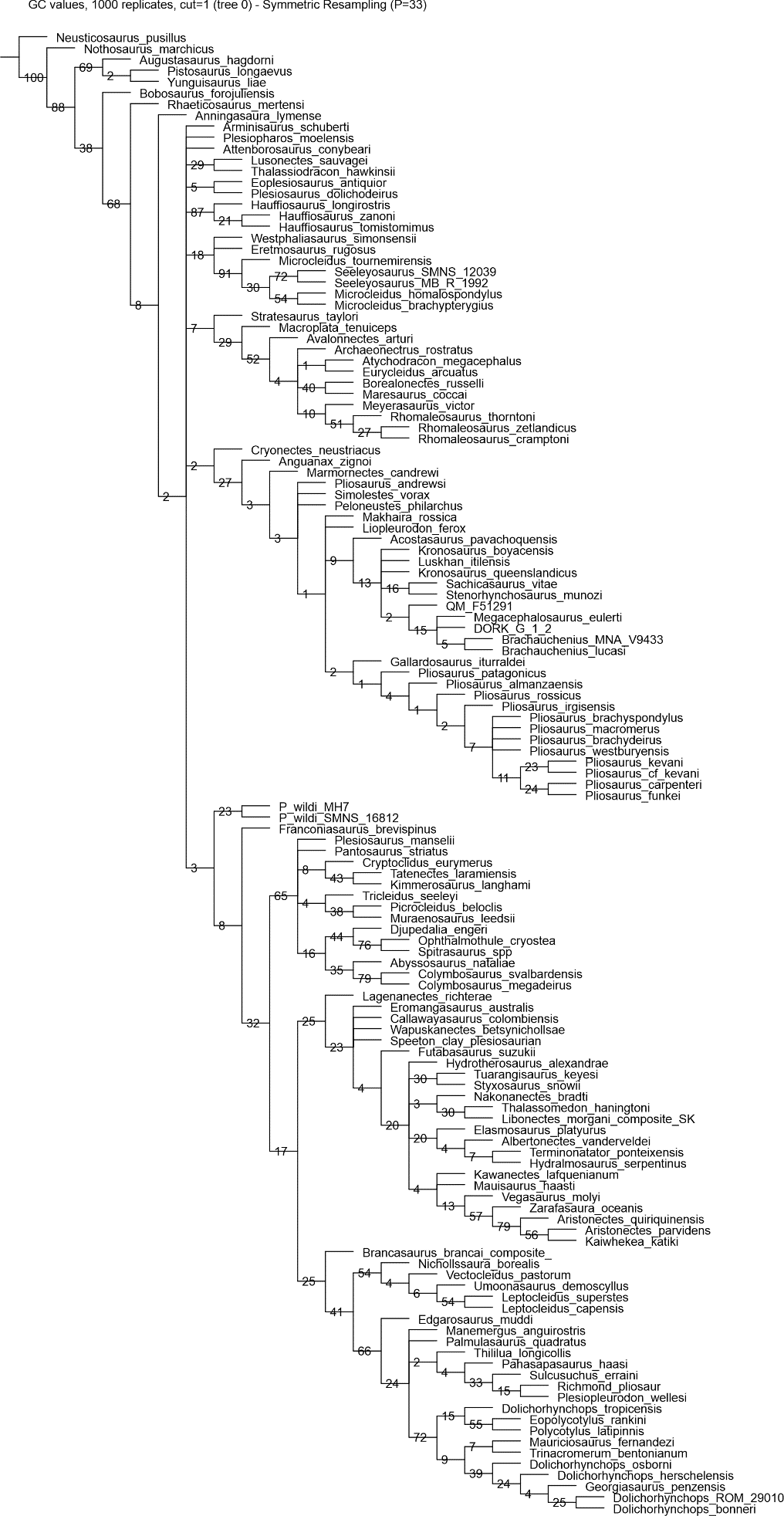

Supplement: Supplemental Information 4 [file peerj-14-20611-s004.docx]
